# How Generative Artificial Intelligence (GAI) reshapes students’ creativity in higher education: A chain path modeling Study from “Exploration–Exploitation” to “Co-Creation–Reflection”

**DOI:** 10.1371/journal.pone.0358186

**Published:** 2026-09-24

**Authors:** Jie Xu, Yixuan Zeng

**Affiliations:** 1 Institute of Creative Arts and Design, UCSI University, Kuala Lumpur, Malaysia; 2 Faculty of Social Sciences and Liberal Arts, UCSI University, Kuala Lumpur, Malaysia; University of Technology Sydney, AUSTRALIA

## Abstract

With the widespread integration of Generative Artificial Intelligence (GAI) into higher education, its relationship with students’ creativity has become a growing focus of scholarly inquiry. Drawing on data from 424 survey responses and in-depth interviews with eight students, this study constructs and examines a chain path model of Exploration–Exploitation–Co-Creation–Reflection to investigate how GAI-assisted learning processes are associated with students’ creativity. Quantitative analyses indicate that exploratory and exploitative behaviors are positively associated with creativity, and these associations are observed alongside perceived cognitive stimulation. Furthermore, co-creation and reflection are significantly associated with flow experience and creative self-efficacy, which are related to students’ creative performance. The qualitative findings complement the quantitative results by showing that students’ engagement in exploration, knowledge utilization, collaborative co-creation, and reflection during GAI-assisted learning is accompanied by cognitive inspiration and psychological engagement. Overall, this study provides insight into how GAI-assisted learning processes may be associated with creativity development in higher education through the interplay of behavioral, cognitive, and psychological mechanisms, offering implications for educational practice and theoretical advancement.

## 1. Introduction

With the rapid advancement of Generative Artificial Intelligence (GAI), its potential applications in education have become increasingly prominent, particularly in relation to students’ creativity in higher education [1,2] GAI not only assists learners in generating diverse creative outputs but may also support exploration and innovative thinking through interactive feedback [3]. However, existing research on how GAI is associated with creativity within educational contexts remains fragmented. Most prior studies have focused on tool efficiency or creative outcomes, while insufficient attention has been paid to the integrative mechanisms encompassing behavioral, cognitive, and psychological dimensions.

The development of creativity relies not only on individuals’ ability to explore new ideas and utilize existing knowledge but also on psychological processes related to collaboration and reflection [4,5] Within the context of higher education, students’ engagement in exploration and exploitation may facilitate cognitive stimulation, while co-creation and reflection may contribute to deeper psychological engagement and creative development. Consequently, integrating the behavioral dimensions of exploration–exploitation with the psychological mechanisms of co-creation–reflection into a chain pathway provides a meaningful framework for examining how GAI-related learning processes are associated with student creativity through multi-level and multi-mechanism pathways.

Nevertheless, three notable gaps persist within the current body of literature. First, prior scholarship rarely distinguishes sequential Exploration–Exploitation and Co-Creation–Reflection behavioral chains, and few integrated chain mediation models have been constructed to unpack their joint effects on student creativity. Second, most existing studies examine cognitive or psychological mediators in isolation, without establishing a complete multi-layer pathway linking learning behaviors, cognitive stimulation, and motivational states. Third, prior work tends to overstate the independent predictive value of single GAI-related behaviors, lacking balanced discussion on their limited individual yet synergistic collective impacts. This study addresses these oversights via a mixed-method chain path analysis.

Building upon this perspective, the present study targets university students and employs a mixed-method design—combining questionnaire surveys with in-depth interviews—to investigate the direct and indirect effects of GAI on creativity through the exploration–exploitation–co-creation–reflection chain mechanism. This study aims to bridge theoretical gaps in the current literature, offer practical guidance for educational innovation, and provide an empirical foundation for designing GAI-assisted learning environments that foster deeper integration between artificial intelligence and creativity cultivation in higher education.

## 2. Theoretical foundation and literature review

### 2.1. Generative AI and the reshaping of creativity in higher education

**Fig 1:**
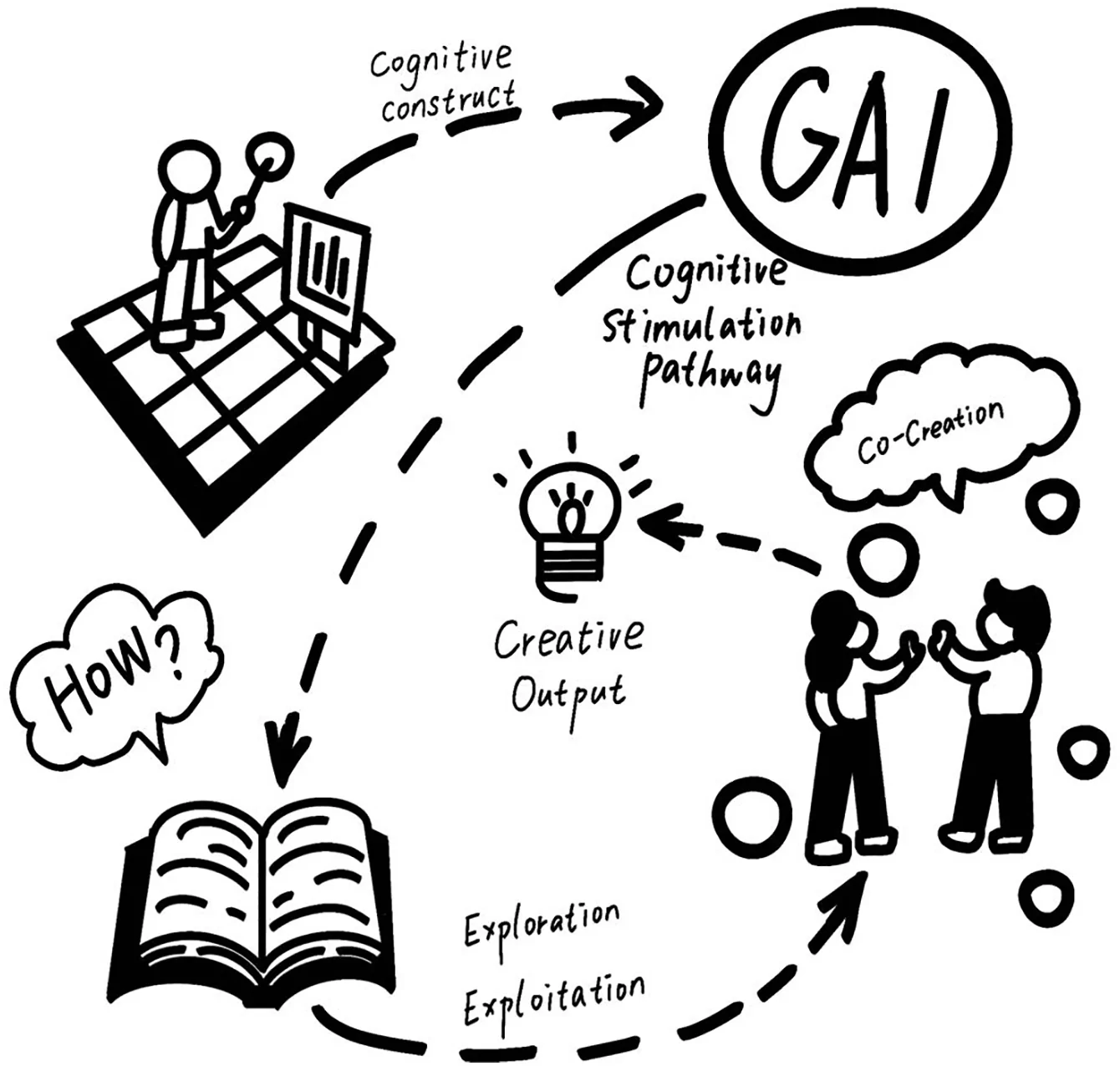
A conceptual model of cognitive and collaborative pathways through which GAI enhances student creativity.

With the rapid development of generative artificial intelligence (GAI) technologies such as ChatGPT and DALL·E, creativity education in higher education is undergoing a profound transformation. As illustrated in Fig 1, traditional approaches to cultivating creativity have primarily relied on teacher guidance, project-based learning, and iterative feedback [4]. The integration of GAI, however, has shifted students’ roles from passive recipients of information or mere producers of content to active co-learners who engage collaboratively and reflectively with AI systems [6]. Recent studies suggest that GAI provides students with extensive creative stimuli, prompting them to undergo more complex cognitive transformation processes during the generation, evaluation, and reconstruction of ideas [7].

In practical teaching contexts, GAI not only expands students’ creative search space but also fosters the development of new metacognitive abilities [8]. An empirical study on AI-assisted academic writing among university students found that after using AI to draft an initial version, students exhibited heightened critical evaluation behaviors—assessing the linguistic style, logical coherence, and factual accuracy of AI-generated texts—and subsequently engaged in rewriting and reflective refinement. This process enabled them to enter a higher level of cognitive self-regulation [9]. Such findings suggest that GAI can function as a “reflection trigger” within instructional design, facilitating a cognitive transition from automatic generation to deliberate selection.

More importantly, GAI redefines the role of the creator in higher education. On one hand, it lowers the technical barriers to creative production, allowing a wider range of students to engage meaningfully in creative expression. On the other hand, it provokes critical reflection on issues of creative authorship and ownership, encouraging students to actively participate in prompt design, output evaluation, and iterative refinement—thereby embodying a human–AI co-creationmechanism [2]. This mechanism not only disrupts the linear structure of traditional creative processes in education but also reshapes the developmental trajectory of creativity itself: from initial exploration and strategic exploitation to collaborative co-creation and self-reflection.

In summary, GAI is reshaping creativity education through two interrelated dimensions. The first is a cognitive activation mechanism, wherein AI-generated content stimulates creative thinking and reflective behaviors. The second is a role restructuring mechanism, through which students evolve from tool users to task decision-makers and creative collaborators. This evolutionary process provides both the empirical foundation and the theoretical rationale for the proposed Exploration–Exploitation–Co-Creation–Reflection chain pathway model, offering a new lens for understanding how GAI redefines creativity cultivation in higher education.

### 2.2. The Theoretical Foundation of the “Exploration–Exploitation” and “Co-Creation–Reflection” Pathway Mechanism

The concept pair of exploration–exploitation originates from organizational learning theory, describing the trade-off individuals face between the exploratory search for new knowledge and the exploitative use of existing knowledge [10,11] Within educational contexts, this framework has been widely employed to explain how learners shift between learning strategies and transfer knowledge, particularly when engaging with complex, open-ended tasks that require balancing divergent thinking and convergent processing [12]. Prior studies have demonstrated that high levels of creativity often depend on an individual’s ability to flexibly alternate between exploration and exploitation—a capacity referred to as learning ambidexterity—which provides an essential theoretical basis for modeling creativity development in GAI-assisted environments [13].

However, the deep integration of GAI into learning processes has transformed not only how knowledge is searched for and applied but also the underlying logic of collaboration—shifting from individual creation toward a human–AI collaborative process of co-creation and reflection [14]. In this mechanism, co-creation transcends the traditional notion of multi-agent cooperation to emphasize dynamic role negotiation and joint task construction within generative interaction. Meanwhile, reflection extends beyond a retrospective evaluation of outcomes to encompass ongoing acts of judgment, selection, and self-regulation throughout the entire creative process [15]. Empirical research further suggests that when students engage in iterative prompting and feedback cycles with GAI, their creative behavior evolves from strategic tool use (exploitation) to metacognitively driven co-creation, continually reconstructing creative intentions and production logic through reflection [14].

**Fig 2:**
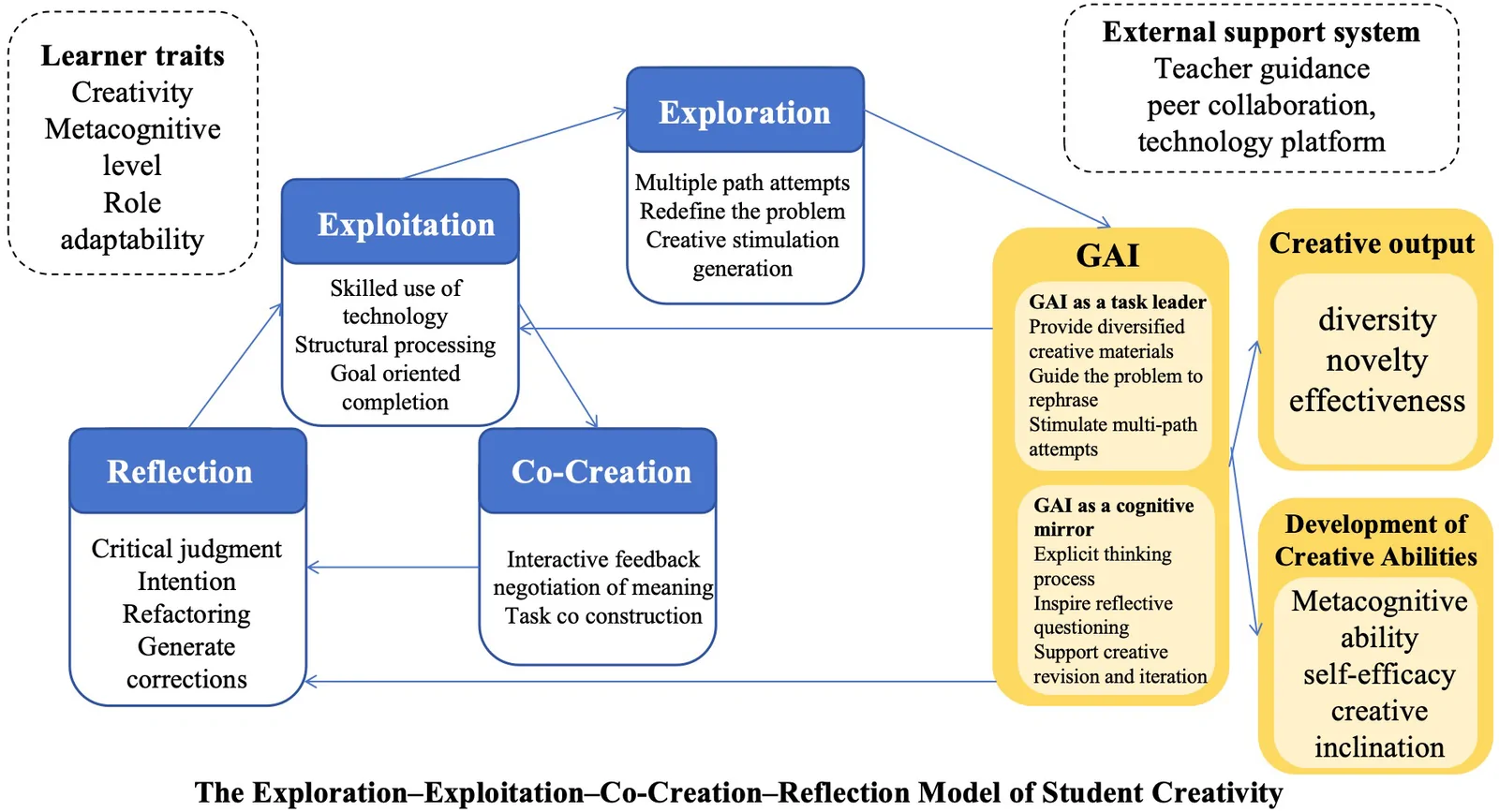
The theoretical pathway model of “Exploration–Exploitation–Co-Creation–Reflection” in GAI-driven creativity development.

Notably, GAI serves a dual function within this dual-pathway mechanism—as both a task guider and a cognitive mirror. On one hand, it provides diverse generative outputs that stimulate students’ creative thinking and guide exploratory attempts in multiple directions. On the other hand, its immediate responses trigger critical evaluation and deep reflection, constructing a continuously evolving cognitive feedback loop [6]. This dynamic process positions the Exploration–Exploitation–Co-Creation–Reflection pathway as not only a cognitively progressive model but also a framework that captures the structural reconfiguration of creative roles and agency. As illustrated in Fig 2, it thus offers an important theoretical lens for understanding the transformation of creativity education driven by GAI.

### 2.3. The mediating mechanisms of GAI-driven student creativity in higher education

In higher education contexts where Generative Artificial Intelligence (GAI) is deeply embedded, the enhancement of students’ creativity does not arise from the technology itself, but from the coordinated activation of multiple psychological and cognitive mediating mechanisms [16,17] Based on the Exploration–Exploitation–Co-Creation–Reflection pathway, this study identifies three key mediators—Perceived Cognitive Stimulation, Flow Experience, and Self-efficacy in Creativity—to elucidate internal psychological pathways linked to associations between GAI and student creativity.

Perceived Cognitive Stimulation, as a cognitive-level mediator, reflects the extent to which GAI enhances the complexity and depth of students’ cognitive processing. Through its capabilities in content generation, problem transformation, and associative expansion, GAI provides diverse and open-ended cognitive inputs that trigger both divergent and convergent thinking. For example, when students use GAI to generate conceptual frameworks of different styles during learning tasks, their cognitive structures are continuously perturbed and reorganized, activating more creative thinking patterns [18,19] Related research shows that students’ perceived cognitive stimulation from interacting with GAI is significantly positively correlated with their creative output [20].

Flow Experience serves as a key affective–motivational mediator. According to Csikszentmihalyi & Csikzentmihaly flow theory [5], flow represents a state of complete immersion in which individuals experience a balance between challenge and skill, accompanied by focused attention and intrinsic enjoyment. In GAI-assisted learning, students often enter this state through iterative prompting, real-time feedback, and multimodal content generation, gaining a heightened sense of control and accomplishment that sustains deep creative engagement [21]. Prior studies indicate that flow experience enhances sustained engagement, deep involvement, and intrinsic motivation during creative activities, which are important psychological conditions supporting creative performance [5].

Self-efficacy in Creativity, as a self-regulatory cognitive mechanism, plays a particularly critical role in human–AI collaborative processes. Bandura [22] proposed that self-efficacy is a core factor influencing goal-setting, persistence, and resilience in specific contexts. Within GAI-assisted learning, students gradually develop a strong belief in their creative capabilities—“I can create”—through repeated feedback, refinement, and iterative generation. This enhanced self-efficacy in creativity not only increases their proactivity and exploratory behavior in subsequent tasks but also significantly improves creative performance in complex problem-solving [23]. Research further shows that students with high self-efficacy in creativity are more likely to draw inspiration from GAI and treat it as a collaborative partner that extends their imaginative and expressive capacities [24].

Based on the dual-path theoretical framework of GAI-enabled creativity, this study imposes theoretically justified boundary constraints on structural paths, rather than arbitrary post-hoc trimming. First, Exploration and Exploitation represent ambidextrous knowledge-seeking and knowledge-utilization behaviors at the cognitive processing level [10]. Their core function lies in expanding and reorganizing information input, which exclusively predicts the cognitive-level mediator Perceived Cognitive Stimulation; these two behavioral constructs lack direct affective or self-regulatory functions, so they do not independently predict immersive Flow Experience or adjustments in creative self-belief (Self-efficacy in Creativity). Second, Co-Creation and Reflection focus on human-AI interactive collaboration and metacognitive self-evaluation, belonging to affective and self-regulatory psychological behaviors. Their core value lies in optimizing task engagement and self-judgment, which only predict Flow Experience and Self-efficacy in Creativity respectively. Collaborative iteration and outcome reflection do not generate new knowledge or cognitive conflict independently, so they cannot directly predict Perceived Cognitive Stimulation. This layered division of cognitive behavior vs. psychological regulation forms the theoretical basis for the structural path constraints of the chain mediation model, rather than data-driven post-hoc adjustment.

Ultimately, GAI’s influence on student creativity is not a unidirectional input–output process. Rather, it emerges from the synergistic interplay among reflection, self-efficacy in creativity, and creative behavior, forming a dynamic triad of reflection–self-regulation–creation. This chain mediation pathway provides a theoretical framework for understanding how GAI transforms creativity education in higher education and offers practical guidance for designing AI-assisted learning systems that are cognitively stimulating and motivationally engaging.

## 3. Research framework and hypotheses

### 3.1. Theoretical model and hypothesis development

Consistent with the dual cognitive-psychological pathway theory proposed in Section 2.3, this study constructs a theoretically bounded chain model: cognitive behaviors (Exploration, Exploitation) only predict Perceived Cognitive Stimulation, while psychological regulatory behaviors (Co-Creation, Reflection) separately predict Flow Experience and Self-efficacy in Creativity.

As Generative Artificial Intelligence (GAI) technologies are increasingly applied in higher education, their impact on students’ learning approaches and creativity development has garnered growing scholarly attention [25]. This study focuses on how students, within GAI-assisted learning and creative processes, gradually activate their intrinsic cognitive potential, affective experiences, and creative output through a four-stage pathway mechanism of Exploration–Exploitation–Co-Creation–Reflection.

**Fig 3:**
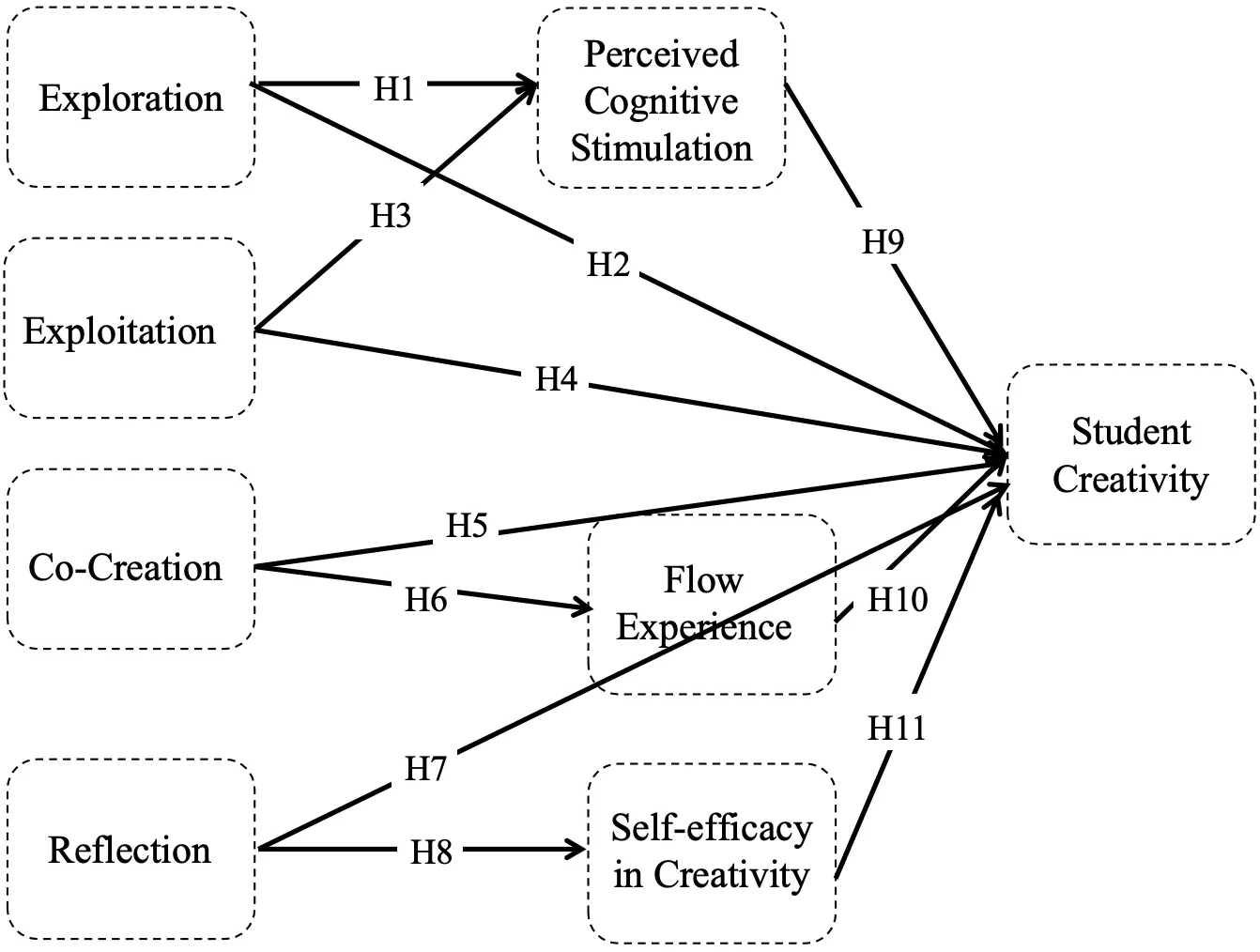
Research Model Diagram.

Building upon prior theoretical research and the literature reviewed in Section 2, this study proposes a student creativity mechanism model, illustrated in Fig 3, and develops a series of testable theoretical hypotheses.

### 3.2. The effects of exploration and exploitation on perceived cognitive stimulation and creativity

Exploration and exploitation represent the behavioral trade-off between novelty-seeking and familiarity-oriented actions when individuals engage in tasks [10]. In GAI-enabled learning environments, exploration manifests as students’ attempts to use AI tools to uncover novel concepts and generate new problems, reflecting high cognitive flexibility and divergent thinking [26]. In contrast, exploitation involves students leveraging AI to organize, summarize, and optimize existing knowledge structures, thereby enhancing learning efficiency and goal-directedness [12].

Both behavioral pathways are likely to trigger students’ perception of task complexity, challenge, and innovation, conceptualized as Perceived Cognitive Stimulation. When students experience information stimuli, cognitive conflict, or the need to reorganize knowledge provided by AI during tasks, they are more likely to develop deep processing motivation and creative thinking tendencies [27]. Based on this reasoning, the following hypotheses are proposed:

H1: Exploration behavior has a positive effect on students’ perceived cognitive stimulation.

H2: Exploration behavior has a positive effect on students’ creativity.

H3: Exploitation behavior has a positive effect on students’ perceived cognitive stimulation.

H4: Exploitation behavior has a positive effect on students’ creativity.

### 3.3. The effects of co-creation and reflection on flow experience and self-efficacy in creativity

Co-Creation emphasizes the generative process of human–AI collaboration. In this stage, students interact continuously with GAI tools, engaging in iterative feedback, refinement, and adjustment, thereby producing learning content or creative outputs that are both original and diverse [28]. This process, characterized by immediate feedback and high interactivity, also facilitates immersive Flow Experience [5], whereby students enter a state of deep engagement under conditions of focused attention, high challenge, and skill-task balance.

Simultaneously, Reflection refers to students’ critical review of AI-generated outcomes and their own learning processes after completing creative tasks. This includes evaluating the effectiveness of content, the logical structure, and their personal contributions. Such reflective behaviors not only enhance cognitive monitoring but also strengthen students’ judgment of their creative capabilities, conceptualized as Self-efficacy in Creativity—a variable widely recognized as a direct predictor of creative intentions and performance [29]. Based on these theoretical considerations, the following hypotheses are proposed:

H5: Co-creation behavior positively influences students’ creativity.
H6: Co-creation behavior positively influences students’ flow experience.
H7: Reflection behavior positively influences students’ creativity.
H8: Reflection behavior positively influences students’ self-efficacy in creativity.

### 3.4. The effects of mediating variables on student creativity

The development of student creativity represents a multidimensional psycho-behavioral construct [30]. In this study, Perceived Cognitive Stimulation, Flow Experience, and Self-efficacy in Creativity are regarded as key mediators, influencing creative outcomes at cognitive, affective, and motivational levels, respectively.

First, when students perceive challenging content and structured problems generated by AI, they are more likely to produce innovative ideas and breakthrough solutions [31]. Second, flow experience enhances enjoyment and concentration during task engagement, providing emotional support and process-driven motivation for complex creative tasks [32]. Finally, self-efficacy in creativity not only affects students’ willingness to engage in creative expression but also determines their persistence in the face of failure and uncertainty [22]. Based on these considerations, the following hypotheses are proposed:

H9: Perceived cognitive stimulation positively influences students’ creativity.
H10: Flow experience positively influences students’ creativity.
H11: Self-efficacy in creativity positively influences students’ creativity.

## 4. Data collection and analysis

### 4.1. Scale design

Based on the Exploration–Exploitation–Co-Creation–Reflection chain pathway framework, this study developed a questionnaire aimed at systematically examining the mechanisms and effects of Generative Artificial Intelligence (GAI) on student creativity in higher education learning contexts. The instrument encompasses nine core dimensions: demographic information, Exploration, Exploitation, Co-Creation, Reflection, Perceived Cognitive Stimulation, Flow Experience, Self-efficacy in Creativity, and Student Creativity, comprising a total of 44 items.

Each construct was adapted from authoritative domestic and international literature [5,10,27,29,33–38] and further tailored to reflect the teaching environment of Chinese higher education and the practical application of mainstream GAI tools, such as ChatGPT, Ernie Bot, Deepseek, and Doubao, thereby enhancing contextual relevance and measurement validity.

Specifically, the Exploration and Exploitation dimensions measure students’ ambidextrous learning behaviors during GAI use. Co-Creation and Reflection capture students’ collaborative creativity and metacognitive regulation in human–AI interaction. Perceived Cognitive Stimulation, Flow Experience, and Self-efficacy in Creativity serve as mediating variables, revealing the cognitive, affective, and belief mechanisms activated during AI interaction. Student Creativityfunctions as the outcome variable, evaluating the overall effect of GAI on learners’ innovative thinking and creative output.

All items were scored using a five-point Likert scale (1 = “Strongly Disagree,” 5 = “Strongly Agree”) to facilitate subsequent structural equation modeling (SEM) analysis. Prior to formal administration, the questionnaire underwent a pilot test to verify clarity and reliability of item expressions. Results indicated that the Cronbach’s α values for all scales exceeded 0.80, and model fit indices were satisfactory (χ²/df < 3, CFI > 0.90, RMSEA < 0.08), demonstrating high reliability and validity of the instrument (Appendix A).

### 4.2. Data collection

This study employed a questionnaire survey to collect data. The survey was administered online via the SoJump platform between April and June 2025, targeting undergraduate, master’s, and doctoral students in Chinese universities with experience using Generative Artificial Intelligence (GAI). A total of 424 valid responses were collected, yielding an effective response rate of 94.2%.

Specifically, the five-item Exploration (Q5–Q9) and five-item Exploitation (Q10–Q14) subscales in this study were adapted from March’s (1991) organizational ambidexterity framework. This framework was originally developed to measure firm-level organizational behaviors rather than individual students’ learning behaviors. To contextualize these constructs within higher education students’ generative artificial intelligence (GAI) learning experiences, all organization-, firm-, and strategic management-related terminology was removed, and each item was reworded to reflect students’ personal learning behaviors and cognitive activities when interacting with GAI. Meanwhile, the original theoretical distinction between novelty-oriented exploration and knowledge-utilization exploitation was retained.

Before the formal survey administration, a pilot study was conducted with 132 valid responses to refine item wording and examine the preliminary psychometric properties of the adapted scales. The pilot participants included undergraduate, master’s, and doctoral students with GAI experience, whose characteristics were consistent with those of the target population in the formal study. Based on the pilot data, an initial confirmatory factor analysis (CFA) was conducted. The results showed that the standardized factor loadings of all items exceeded 0.70, Cronbach’s α coefficients ranged from 0.81 to 0.88, composite reliability (CR) values exceeded 0.70, and average variance extracted (AVE) values were above 0.50. These findings indicated that the adapted Exploration and Exploitation scales demonstrated acceptable preliminary reliability and convergent validity.

Based on participant feedback, minor wording modifications were made to three Exploration items and two Exploitation items to improve clarity and contextual appropriateness, while no items were removed. The decision to retain all five items for each construct was supported by both the pilot statistical results and the theoretical foundation of ambidextrous learning. Following these refinements, the finalized questionnaire was administered for large-scale formal data collection.

Prior to data collection, all participants were fully informed of the purpose of the study, the procedures involved, and their rights as research participants. Informed consent was obtained from all participants before they completed the questionnaire and participated in the interviews. Participation was entirely voluntary, and participants were informed that they could withdraw from the study at any stage without any negative consequences. All data collection and processing procedures adhered to the principles of anonymity and confidentiality to ensure the privacy and security of participants’ information.

This study obtained formal ethical exemption from the Faculty of Social Sciences and Liberal Arts, UCSI University, in compliance with the university’s research ethics procedures and relevant international educational research ethics guidelines, including those issued by the British Educational Research Association (BERA). UCSI University has a comprehensive institutional ethics review framework. For minimal-risk educational research conducted in regular academic settings, ethical reviews are conducted through authorized faculty-level ethics panels. This project was assessed and granted an ethical waiver by the faculty-level ethics panel. It was classified as minimal risk because participation was entirely voluntary, informed consent was obtained from all participants, and all research data were anonymized. The study did not collect sensitive personal information, involve vulnerable populations, conduct clinical interventions, or adopt procedures beyond routine educational activities. The approving body was the Faculty of Social Sciences and Liberal Arts, UCSI University. An official ethical exemption document with signatures and institutional stamps was issued and retained by the research team.

Regarding gender distribution, 218 respondents were male (51.4%) and 206 were female (48.6%). In terms of academic level, 102 were undergraduates (24.1%), 163 were master’s students (38.4%), and 159 were doctoral students (37.5%). Regarding GAI usage experience, 21 respondents (5.0%) had used GAI for less than three months, 16 (3.8%) for three to six months, 178 (42.0%) for six to twelve months, 162 (38.2%) for one to two years, and 47 (11.1%) for more than two years. In terms of tool usage, the most frequently used GAI platform was ChatGPT (367 respondents, 86.6%), followed by Deepseek (287, 67.7%), Wenxin Yiyan/Ernie Bot (212, 50.0%), and Doubao (203, 47.9%). In terms of disciplinary backgrounds, all 424 survey respondents were divided into three consistent clusters: Arts & Humanities, Social Sciences, and STEM & Medicine. Specifically, 168 participants (39.6%) belonged to Arts & Humanities majors, 131 (30.9%) to Social Sciences, and 125 (29.5%) to STEM & Medicine disciplines. Overall, the sample structure was relatively balanced, providing strong representativeness and reliability for the study.

Structural Equation Modeling (SEM) was employed as the primary analytical method. Accordingly, the sample size was strictly determined based on SEM requirements. According to Hair et al. [39], the sample size in SEM should be proportional to model complexity, with a general recommendation of 5–10 times the number of observed variables to ensure stability of estimates and model fit. Barrett [40] noted that while larger samples can improve statistical power, sample sizes exceeding 500 may lead to chi-square statistics being overly sensitive, potentially distorting model fit indices or significance judgments.

Considering that the present study’s model includes 44 observed variables and 8 latent constructs, the actual sample size of 424 corresponds to approximately 9.6 times the number of observed indicators, fully meeting theoretical SEM guidelines. This sample size ensures stable estimation of model parameters and statistical significance of path coefficients while avoiding potential bias caused by excessive sample size, thus providing strong representativeness and statistical adequacy.

### 4.3. Reliability and validity analysis

The questionnaire data in this study were collected through learner self-reports, primarily reflecting students’ subjective perceptions of their Generative Artificial Intelligence (GAI) learning experiences. Given that single-source data may lead to common method variance (CMV), control measures were implemented during the survey design. Following the recommendations of Podsakoff et al. [41] and Shiau et al. [42], items for different latent constructs were distributed across separate pages, with buffer instructions and randomized item order introduced to reduce response biases and order effects, thereby mitigating the risk of CMV.

Beyond the procedural remedies for common method variance (CMV), we further conducted Harman’s single-factor test to empirically evaluate the potential method bias. The results of the unrotated exploratory factor analysis showed that the variance explained by the first single factor was 23.06%, which was well below the critical threshold of 40%. Nevertheless, Harman’s single-factor test serves only as a preliminary screening tool and cannot fully rule out residual common-method-bias effects. This finding provides preliminary evidence that severe common method variance is unlikely to dominate our dataset.

Additionally, all average variance extracted (AVE) values clustered in a narrow range (0.532–0.581) and all correlations in Table 2 were positive. We clarify that this pattern is inherent to the theoretical relationships among the constructs in our GAI-enabled creative learning framework, rather than an artifact caused by common method bias. Taken together with our procedural controls and Harman’s single-factor outcomes, these observations alleviate major concerns regarding large-scale distortion stemming from common method variance, although minor residual bias cannot be completely excluded.

Regarding reliability, confirmatory factor analysis (CFA) results from Structural Equation Modeling (SEM) (see Table 1) indicated that composite reliability (CR) for all latent constructs ranged from 0.850 to 0.874, exceeding the 0.70 threshold recommended by Fornell and Larcker (1981), suggesting good internal consistency. Standardized factor loadings for all items were significant and above 0.70 (p < 0.001), and squared multiple correlations (SMC) values fell within a reasonable range, demonstrating stable and reliable measurement indicators.Convergent validity results showed that the average variance extracted (AVE) for all latent constructs exceeded 0.50 (ranging from 0.532 to 0.581), meeting the criterion for adequate convergent validity. Among these, Exploration (AVE = 0.581, CR = 0.874) and Self-efficacy in Creativity (AVE = 0.581, CR = 0.874) scored highest, indicating strong internal consistency among their items. Flow Experience (AVE = 0.575, CR = 0.871) and Co-Creation (AVE = 0.546, CR = 0.857) also demonstrated stable measurement properties.

**Table 1:** Convergent validity test table.

Dimensions	Question	Significance Estimate				Item Reliability		Component Reliability	Convergent Validity
		UnStd.	S.E.	z-value	P	Std.	SMC	CR	AVE
Exploration	Q5	1				0.792	0.627	0.874	0.581
	Q6	0.969	0.059	16.492	***	0.772	0.596		
	Q7	0.914	0.06	15.328	***	0.725	0.526		
	Q8	0.957	0.06	15.999	***	0.752	0.566		
	Q9	0.956	0.058	16.421	***	0.769	0.591		
Exploitation	Q10	1				0.712	0.507	0.85	0.532
	Q11	0.914	0.068	13.448	***	0.72	0.518		
	Q12	0.995	0.07	14.256	***	0.768	0.59		
	Q13	0.923	0.067	13.714	***	0.735	0.54		
	Q14	0.898	0.067	13.32	***	0.712	0.507		
Co-Creation	Q15	1				0.754	0.569	0.857	0.546
	Q16	1.019	0.068	14.92	***	0.753	0.567		
	Q17	0.981	0.065	14.985	***	0.756	0.572		
	Q18	0.881	0.063	13.944	***	0.705	0.497		
	Q19	0.917	0.064	14.308	***	0.723	0.523		
Reflection	Q20	1				0.797	0.635	0.868	0.57
	Q21	0.978	0.06	16.254	***	0.767	0.588		
	Q22	0.915	0.059	15.425	***	0.732	0.536		
	Q23	0.836	0.057	14.548	***	0.696	0.484		
	Q24	0.994	0.06	16.539	***	0.778	0.605		
Perceived Cognitive Stimulation	Q25	1				0.749	0.561	0.853	0.538
	Q26	0.926	0.066	13.949	***	0.719	0.517		
	Q27	0.976	0.069	14.187	***	0.731	0.534		
	Q28	0.953	0.069	13.888	***	0.716	0.513		
	Q29	0.978	0.067	14.582	***	0.752	0.566		
Flow Experience	Q30	1				0.748	0.56	0.871	0.575
	Q31	0.971	0.065	14.913	***	0.752	0.566		
	Q32	1.03	0.065	15.843	***	0.799	0.638		
	Q33	0.992	0.065	15.36	***	0.774	0.599		
	Q34	0.913	0.064	14.15	***	0.714	0.51		
Self-efficacy in Creativity	Q35	1				0.749	0.561	0.874	0.581
	Q36	1.05	0.068	15.406	***	0.772	0.596		
	Q37	1.07	0.067	15.846	***	0.795	0.632		
	Q38	1.027	0.07	14.703	***	0.738	0.545		
	Q39	1.008	0.067	15.028	***	0.754	0.569		
Student Creativity	Q40	1				0.744	0.554	0.867	0.567
	Q41	1.028	0.066	15.473	***	0.783	0.613		
	Q42	1.024	0.067	15.253	***	0.772	0.596		
	Q43	1.006	0.067	14.997	***	0.759	0.576		
	Q44	0.915	0.066	13.905	***	0.705	0.497		

***p < 0.001

Overall, the questionnaire demonstrated high levels of reliability and convergent validity, effectively capturing the latent characteristics of key variables in GAI-assisted learning, thereby providing a robust foundation for subsequent model fitting and path analysis.

To validate the measurement properties of the questionnaire, this study assessed both convergent validity and discriminant validity, based on measurement results obtained from Structural Equation Modeling (SEM) and following the criteria proposed by Fornell and Larcker [43].

Convergent validity was evaluated using the average variance extracted (AVE) for each latent construct, with higher AVE values indicating that a construct captures more meaningful information from its indicators. Generally, an AVE greater than 0.50 is considered indicative of adequate convergent validity. In this study, all latent constructs had AVE values exceeding this threshold, demonstrating that each construct effectively captured the information from its corresponding items and exhibited strong internal consistency.

Discriminant validity was assessed to examine the distinctiveness of each latent construct. The Fornell-Larcker criterionwas applied, which requires that the square root of a construct’s AVE be greater than its correlations with other constructs. As shown in Table 2, the diagonal values represent the square roots of AVE for each construct, all of which exceed their correlations with other constructs. For instance, the highest correlation of Exploration with another construct is 0.575 (with Exploitation), whereas its AVE square root is 0.762, well above the correlation. Similarly, the AVE square root of Student Creativity is 0.753, exceeding all its correlations with other constructs. Other constructs, including Reflection (0.755), Co-Creation (0.739), Exploitation (0.729), Flow Experience (0.758), Self-efficacy in Creativity (0.762), and Perceived Cognitive Stimulation (0.733), all satisfy the discriminant validity criterion. Overall, these results indicate good discriminant validity among latent constructs, demonstrating that the questionnaire effectively measures distinct psychological and behavioral dimensions.

**Table 2:** Discriminant validity table.

	Convergent Validity	Discriminant validity							
	AVE	Reflection	Co-Creation	Exploitation	Exploration	Flow Experience	Self-efficacy in Creativity	Perceived Cognitive Stimulation	Student Creativity
Reflection	0.57	**0.755**							
Co-Creation	0.546	0.254	**0.739**						
Exploitation	0.532	0.126	0.466	**0.729**					
Exploration	0.581	0.149	0.303	0.575	**0.762**				
Flow Experience	0.575	0.124	0.486	0.226	0.147	**0.758**			
Self-efficacy in Creativity	0.581	0.453	0.115	0.057	0.067	0.056	**0.762**		
Perceived Cognitive Stimulation	0.538	0.069	0.184	0.374	0.409	0.09	0.031	**0.733**	
Student Creativity	0.567	0.286	0.434	0.437	0.429	0.299	0.217	0.313	**0.753**

**Note**: The bold numbers on the diagonal are the square root of AVE, and the lower triangle is the Pearson correlation coefficient of the variables

Prior research notes that the Fornell-Larcker criterion has limited reliability for evaluating discriminant validity when constructs share similar theoretical connotations and high intercorrelations [44]. Therefore, this study further adopted the Heterotrait-Monotrait (HTMT) ratio as a supplementary and more rigorous test of discriminant validity. Consistent with the recommended judgment rules, we set a stricter threshold of 0.85 for theoretically unrelated constructs and a relaxed cut-off value of 0.90 for two conceptually analogous constructs (Exploration and Exploitation). The HTMT value between Exploration and Exploitation reached 0.8951, which was below the 0.90 standard for similar ambidexterity constructs. All remaining pairwise HTMT ratios across the other six latent variables ranged from 0.2519 to 0.5218, all far lower than the 0.85 critical value. Collectively, the HTMT results further corroborate adequate discriminant validity of all measurement constructs.

### 4.4. Model fit assessment

The fit of the theoretical model was evaluated using Structural Equation Modeling (SEM) (Table 3), and the results indicated that the overall model fit was satisfactory. Specifically, the chi-square statistic was CMIN = 992.826 with DF = 723, yielding a CMIN/DF ratio of 1.373, which is well below the recommended threshold of 3, indicating a relatively good chi-square fit. The Goodness-of-Fit Index (GFI) = 0.897 and Adjusted Goodness-of-Fit Index (AGFI) = 0.884 both fall within acceptable ranges, with GFI approaching the threshold for a good fit and AGFI slightly below 0.90, yet still reflecting an adequate overall model fit.

**Table 3:** Model fit indicators.

Index	Model Value Indicator	Standard	Conclusion	Standard Source
CMID	992.826	The smaller the better		
DF	723	The smaller the better		
CMID/DF	1.373	<3	Good fit	[48]
GFI	0.897	>0.8 Acceptable; > 0.9 Good fit	Acceptable	[47]
AGFI	0.884	>0.8 Acceptable; > 0.9 Good fit	Acceptable	[49]
CFI	0.966	>0.9	Good fit	[47]
TLI (NNFI)	0.964	>0.9	Good fit	[45]
RMSEA	0.03	<0.08	Good fit	[45]
SRMR	0.057	<0.08	Good fit	[46]

Regarding incremental fit indices, the Comparative Fit Index (CFI) = 0.966 and Tucker-Lewis Index (TLI/NNFI) = 0.964 both exceed 0.90, suggesting excellent fit from a comparative perspective. The approximate fit indices, Root Mean Square Error of Approximation (RMSEA) = 0.03 and Standardized Root Mean Square Residual (SRMR) = 0.057, are below the 0.08 threshold, further supporting satisfactory model fit.In summary, the Exploration–Exploitation–Co-Creation–Reflection chain mediation model constructed in this study adequately matches the empirical data, demonstrating robust structural stability and explanatory power.

This study emphasizes reporting seven fit indices—CMIN/DF, GFI, AGFI, CFI, TLI, RMSEA, and SRMR—because they collectively provide a comprehensive assessment of model fit from multiple perspectives, including chi-square fit, residual fit, comparative fit, and approximate error [45–47].

All structural equation model estimations were performed using AMOS 26.0 with the Maximum Likelihood (ML) estimator. Before model fitting, multivariate normality screening was conducted for all observed indicator variables, and mild multivariate non-normality was detected. Therefore, bias-corrected bootstrapping with 5,000 random resamples was employed to obtain robust estimates of all indirect and chain mediation effects. Regarding missing data handling, the 44-item Likert questionnaire contained less than 0.8% random missing responses across all items. Full Information Maximum Likelihood (FIML) was adopted to handle missing values rather than listwise deletion, preserving the full valid sample size of N = 424 and avoiding potential loss of statistical power.

### 4.5. Structural model validation

**Fig 4:**
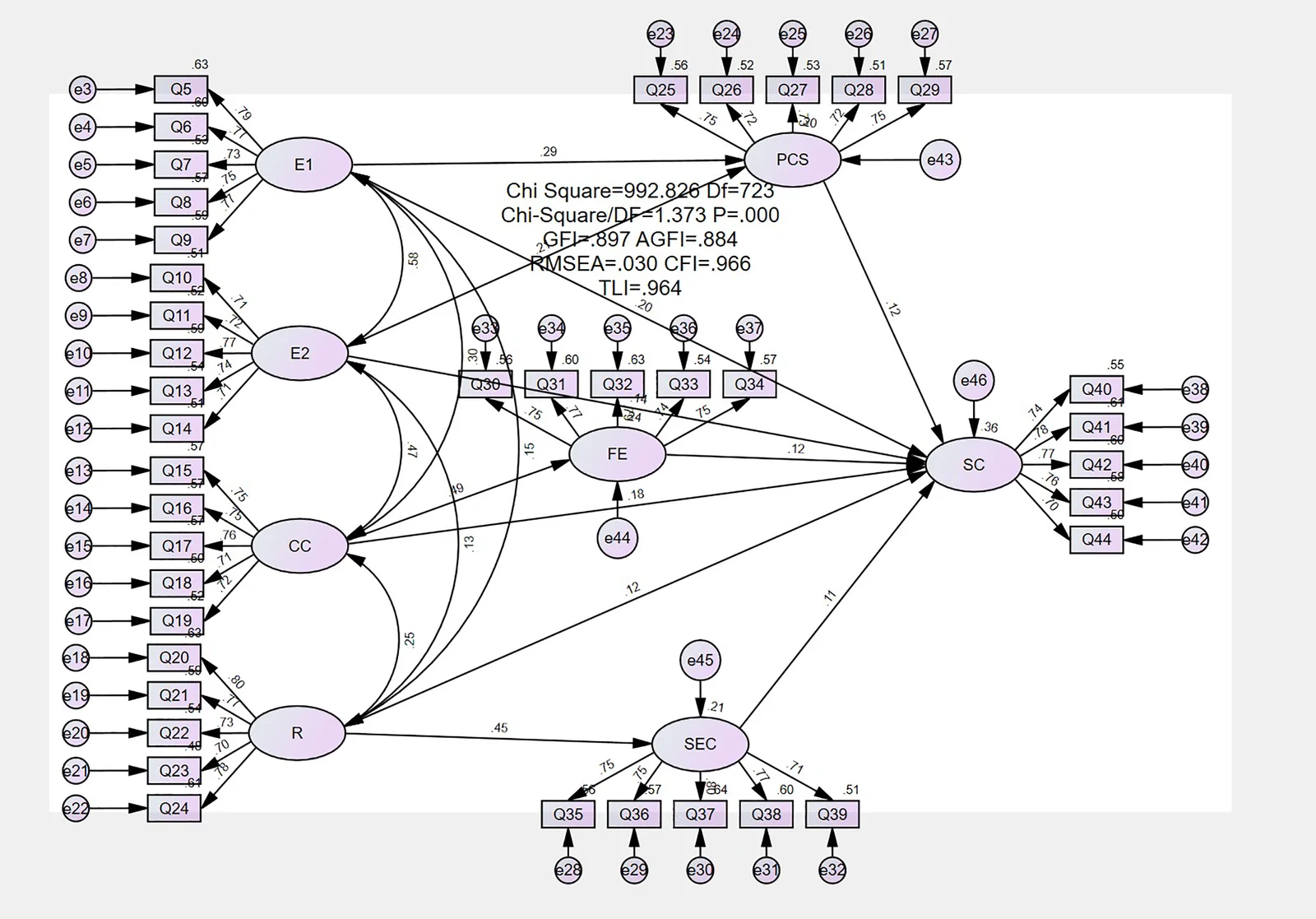
Structural equation model diagram. Note: PCS = Perceived Cognitive Stimulation; FE = Flow Experience; SEC = Self-efficacy in Creativity; SC = Student Creativity; E1 = Exploration; E2 = Exploitation; CC = Co-Creation; R = Reflection.

This study employed Amos 26.0 to validate the Structural Equation Model (SEM), estimating path coefficients and the variance explained (R^2^) for latent constructs, as illustrated in Fig 4. The model testing results indicate that all 11 proposed hypotheses (H1–H11) reached statistical significance, receiving empirical support. Detailed path effects are presented in Table 4.

**Table 4:** Model path analysis results.

	UnStd.	S.E.	C.R.	P	Std.	R^2^
Exploration --- > Perceived Cognitive Stimulation	0.275	0.066	4.166	***	0.289	0.196
Exploitation --- > Perceived Cognitive Stimulation	0.198	0.066	2.984	0.003	0.207	
Co-Creation --- > Flow Experience	0.478	0.058	8.262	***	0.486	0.236
Reflection --- > Self-efficacy in Creativity	0.438	0.055	7.901	***	0.453	0.206
Perceived Cognitive Stimulation --- > Student Creativity	0.123	0.056	2.212	0.027	0.121	0.359
Flow Experience --- > Student Creativity	0.118	0.057	2.06	0.039	0.116	
Self-efficacy in Creativity --- > Student Creativity	0.106	0.053	2.012	0.044	0.11	
Reflection --- > Student Creativity	0.111	0.053	2.091	0.037	0.119	
Exploration --- > Student Creativity	0.192	0.063	3.07	0.002	0.199	
Exploitation --- > Student Creativity	0.139	0.068	2.054	0.04	0.144	
Co-Creation --- > Student Creativity	0.185	0.066	2.78	0.005	0.185	

This study adopts a theoretically constrained structural model instead of a saturated full model. The path separation design strictly follows the dual cognitive-psychological creativity formation mechanism derived from literature review, with clear theoretical boundaries between cognitive ambidextrous behaviors and metacognitive collaborative behaviors, which avoids overfitting caused by meaningless cross-variable paths. The theoretical rationales for all path constraints are fully elaborated in Section 2.3.

Specifically, Exploration, Exploitation, Co-Creation, and Reflection all positively predicts Perceived Cognitive Stimulation, Flow Experience, and Self-efficacy in Creativity. Both Exploration and Exploitation further demonstrated direct positive impacts on Student Creativity (H1–H4). Additionally, Co-Creation positively influenced Student Creativity and Flow Experience (H5–H6), while Reflection positively affected Student Creativity and Self-efficacy in Creativity (H7–H8). Finally, Perceived Cognitive Stimulation, Flow Experience, and Self-efficacy in Creativity each had significant positive effects on Student Creativity (H9–H11).

Regarding the strength of path effects (Table 4), Exploration exhibited a standardized coefficient of 0.289 (p < 0.001) on Perceived Cognitive Stimulation, while Exploitation showed a standardized coefficient of 0.207 (p = 0.003), indicating that Exploration is a stronger positive predictor of cognitive stimulation than Exploitation. Co-Creation had the most pronounced effect on Flow Experience (Std. = 0.486, p < 0.001), and Reflection had a comparatively strong impact on Self-efficacy in Creativity (Std. = 0.453, p < 0.001).

With respect to the outcome variable, Exploration and Exploitation had standardized coefficients of 0.199 and 0.144 on Student Creativity (p < 0.01), suggesting that Exploration is a stronger positive predictor of student creativity relative to Exploitation. Furthermore, Perceived Cognitive Stimulation, Flow Experience, and Self-efficacy in Creativity all exerted positive and significant effects on Student Creativity (Std. = 0.121, 0.116, and 0.110, respectively; p < 0.05). Both Reflection (0.119, p = 0.037) and Co-Creation (0.185, p = 0.005) also had significant positive effects on creativity.

The R^2^ values indicate that the model demonstrates satisfactory explanatory power for the core outcome variables: Perceived Cognitive Stimulation (R^2^ = 0.196), Flow Experience (R^2^ = 0.236), Self-efficacy in Creativity (R^2^ = 0.206), and Student Creativity (R^2^ = 0.359). These results suggest that the model adequately accounts for the variance in key behavioral and outcome variables in GAI-assisted learning. In summary, the proposed research model exhibits structural stability and clearly defined path relationships, effectively revealing the core mechanisms through which generative artificial intelligence reshapes student creativity in higher education.

It should be noted that the R^2^ values of the three mediating variables ranged from 0.196 to 0.236, while Student Creativity showed an R^2^ value of 0.359. We acknowledge that these values indicate that a considerable proportion of variance remains unexplained. However, in educational and psychological SEM research, latent psychological constructs such as perceived cognitive stimulation, flow experience, and self-efficacy in creativity are typically influenced by multiple individual, contextual, and environmental factors that cannot always be fully incorporated into a single theoretical model. The current framework focuses specifically on the cognitive and psychological mechanisms through which GAI-supported learning behaviors contribute to creativity development. Other potential antecedents, including personality traits, prior creative experience, instructor support, disciplinary characteristics, and broader learning environments, were beyond the scope of the present study and may account for additional variance.

#### 4.5.1. Bootstrapped chain mediation analysis.

To formally verify the proposed chain mediation mechanisms and comply with PLOS ONE methodological standards as well as the classic mediation analysis framework proposed by Hayes & Little [50] and Preacher & Hayes [51], this study conducted bias-corrected bootstrapped mediation tests with 5,000 random resamples. Merely examining significant direct path coefficients cannot statistically validate chain mediation effects; formal estimation of indirect effects with bootstrapped confidence intervals is mandatory to confirm sequential mediation hypotheses. A chain mediation pathway is defined as statistically significant when its 95% bias-corrected confidence interval does not contain zero. All four theoretically hypothesized sequential indirect pathways were extracted from AMOS bootstrap output. The standardized indirect effect of Exploration on student creativity via Perceived Cognitive Stimulation was 0.058 (95% BC CI = [0.017, 0.123], p = 0.004). The indirect effect of Exploitation on creativity through Perceived Cognitive Stimulation was 0.041 (95% BC CI = [0.012, 0.094], p = 0.004). For the psychological mediation chain, Co-Creation exerted a significant indirect effect of 0.070 on student creativity through Flow Experience (95% BC CI = [0.003, 0.146], p = 0.041), while Reflection indirectly predicted creativity with an indirect effect of 0.065 via Self-efficacy in Creativity (95% BC CI = [0.002, 0.134], p = 0.041). All bootstrapped standardized indirect effect results, corresponding bias-corrected confidence intervals and p-values are summarized in the standalone Table 5.

**Table 5:** Bootstrapped chain mediation analysis.

Mediated Chain Pathway	Standardized Indirect Effect	Bias-Corrected 95% CI (Lower, Upper)	p-value	Significance
Exploration → Perceived Cognitive Stimulation → Student Creativity	0.058	(0.017, 0.123)	0.004	Significant
Exploitation → Perceived Cognitive Stimulation → Student Creativity	0.041	(0.012, 0.094)	0.004	Significant
Co-Creation → Flow Experience → Student Creativity	0.07	(0.003, 0.146)	0.041	Significant
Reflection → Self-efficacy in Creativity → Student Creativity	0.065	(0.002, 0.134)	0.041	Significant

Note: Bootstrap resampling = 5,000 times; mediation effect is statistically significant if the 95% bias-corrected confidence interval excludes zero.

### 4.6. Follow-up qualitative analysis

To further complement the quantitative findings and gain deeper insights into the mechanisms revealed by the structural model, this study conducted semi-structured interviews with eight purposefully selected students from the survey sample (N = 424), representing diverse disciplinary backgrounds and varying levels of AI experience. The interview protocol (Appendix B) focused on the eight core constructs of the model: demographic information, Exploration, Exploitation, Co-Creation, Reflection, Perceived Cognitive Stimulation, Flow Experience, Self-efficacy in Creativity, and Student Creativity. Each interview lasted approximately 25 minutes and was transcribed verbatim.

This study adopted stratified purposive sampling with three clear pre-defined screening standards to realize multi-dimensional sample diversity:

1 Academic stage stratification: We covered undergraduates, master’s students, and doctoral students to match the demographic distribution of the 424 quantitative respondents. Among eight interviewees, there were 2 undergraduates, 3 master’s students, and 3 doctoral students.
2 GAI usage duration stratification: Two mainstream experience tiers from the questionnaire were included: 6–12 months (2 participants) and 1–2 years (6 participants).
3 Disciplinary diversity operationalization: All interviewees were divided into three disciplinary clusters: Arts & Humanities, Social Sciences, and STEM & Medicine, covering education, visual design, music, sociology, clinical medicine and material engineering to guarantee cross-major perspectives.

Beyond these demographic stratifications, we also selected participants with contrasting high and low scores on Exploration and Exploitation scales from the quantitative dataset to capture diverse ambidextrous learning experiences.

To judge data sufficiency, we followed the thematic saturation criterion proposed by Braun & Clarke[52]. After coding each transcript one by one, new core sub-themes kept emerging in the first five interviews. Starting from the sixth participant, no novel thematic dimensions related to our chain model appeared. The 7th and 8th interviewees only repeated existing viewpoints without generating new insights, so we stopped recruiting more participants after eight interviews. Detailed background information of all interviewees is listed in the Table 6.

**Table 6:** Demographic characteristics of eight interview participants.

ID	Gender	Academic Stage	Disciplinary Cluster	Specific Major	Duration of GAI Use	Commonly Used GAI Tools
P1	Male	Undergraduate	Arts & Humanities	Education	6–12 months	ChatGPT, DeepSeek, Wenxin Yiyan
P2	Female	Undergraduate	Arts & Humanities	Visual Communication Design	1–2 years	ChatGPT, Midjourney, Wenxin Yiyan
P3	Male	Master’s	STEM & Medicine	Clinical Medicine	1–2 years	ChatGPT, DeepSeek, Wenxin Yiyan
P4	Female	Master’s	Social Sciences	Sociology	6–12 months	ChatGPT, DeepSeek, Wenxin Yiyan
P5	Male	Master’s	Arts & Humanities	Music Composition	1–2 years	ChatGPT, DeepSeek, Wenxin Yiyan, Suno
P6	Male	Doctoral	Arts & Humanities	Educational Research	1–2 years	ChatGPT, Wenxin Yiyan
P7	Female	Doctoral	Arts & Humanities	Educational Philosophy	1–2 years	ChatGPT, DeepSeek, Wenxin Yiyan
P8	Female	Doctoral	STEM & Medicine	Material Engineering	1–2 years	ChatGPT, DeepSeek, Wenxin Yiyan

Data were analyzed using Thematic Analysis as proposed by Braun and Clarke [52], employing the key variables of the quantitative model as the initial coding framework while inductively refining themes from the interview transcripts (Table 7). To enhance the reliability and validity of the analysis, researcher triangulation and member checking were implemented throughout the coding process.

**Table 7:** Qualitative coding results.

Main Construct	Sub-theme	Description	Representative Quote
Exploration	New Idea Generation	Students generate unexpected thought paths or perspectives through AI	“AI often provides unexpected answers, which makes me think about the problem from different angles.”
	Knowledge Expansion	Students use AI to access concepts and knowledge they have not encountered before	“I use AI to explore new theories, which allows me to see a broader range of ideas.”
Exploitation	Structural Optimization	Students use AI to improve the structure or logic of existing outputs	“AI helped me reorganize the structure of my paper more clearly.”
	Efficiency Enhancement	AI improves students’ learning efficiency and quality of outputs	“Using AI saved me a lot of time, allowing me to focus more on core issues.”
Co-creation	Human–AI Collaboration	Students treat AI as a co-creator, jointly generating outputs	“AI is like a collaborator; I work with it to refine my plans.”
	Iterative Improvement	Students enhance output quality through repeated prompts and feedback	“I keep modifying the prompts until the results are satisfactory.”
Reflection	Critical Evaluation	Students reflect on the limitations and appropriateness of AI outputs	“When AI’s answers are not ideal, I think about what went wrong and how I can improve it.”
	Learning Role Reconstruction	Students reflect on their own learning responsibilities and methods through AI use	“AI made me realize that I cannot rely on it completely; I need to take a more proactive role.”
Perceived Cognitive Stimulation	Cognitive Disruption	AI outputs trigger cognitive conflicts and reprocessing	“AI gave answers that differed from my expectations, prompting me to investigate why.”
	Interest Stimulation	Interaction with AI increases learning interest and cognitive engagement	“AI’s answers are interesting, which makes me want to keep asking questions.”
Flow Experience	Focused Engagement	Students enter a flow state while interacting with AI	“When adjusting prompts, I often lose track of time.”
	Challenge and Control	Students gain a sense of control while attempting to adjust AI responses	“I enjoy adjusting prompts, feeling that I am directing my learning.”
Self-efficacy in Creativity	Creative Confidence	Students’ belief in their ability to innovate is enhanced with AI support	“AI makes me feel that I can also come up with new ideas.”
	Problem-Solving Confidence	Students feel more confident tackling difficult problems	“I used to be afraid I couldn’t write, but now I dare to try new topics.”
Student Creativity	Creative Output	Students perceive AI as improving the originality and quality of their work	“AI helped me refine my work, making it more creative.”
	Idea Inspiration	AI promotes divergent thinking and conceptual recombination	“AI inspires me to explain the same problem from different perspectives.”

To guarantee the reliability and consistency of thematic coding, two independent researchers separately coded all full interview transcripts independently and without prior discussion. Cohen’s Kappa coefficient was calculated to assess inter-rater reliability, yielding a value of 0.84, indicating almost perfect agreement between the two coders. Minor discrepancies in coding judgements were resolved through joint re-reading of the original interview transcripts and discussion between the two researchers before finalising the thematic categories.

The interview findings indicate that Exploration helps students break through existing cognitive limitations and triggers cognitive disruption through the discovery of novel ideas and knowledge extension, thereby enhancing cognitive activation. For instance, one student noted: *“AI often provides unexpected answers, prompting me to consider problems from different perspectives.”* Exploitation, in contrast, primarily manifests as structural optimization and efficiency enhancement, assisting students in organizing and processing knowledge more effectively, thereby improving the quality of learning outcomes: *“AI helped me reorganize the structure of my paper more clearly, saving me a lot of time.”*

During the creative generation process, Co-Creation was particularly critical. Students treated AI as a collaborative partner, iteratively refining outputs through repeated prompts and feedback, resulting in high-quality outcomes and immersive flow experiences: *“AI is like a collaborator; I refine the plan together with it. Continuously adjusting the prompts keeps me fully immersed.”* Reflection, on the other hand, reinforced metacognition and learning self-regulation, prompting students to critically evaluate AI outputs and reconstruct their own learning roles, thereby enhancing Self-efficacy in Creativity: *“AI made me realize that I cannot rely on it entirely and need to take a more proactive role.”*

Furthermore, Perceived Cognitive Stimulation, Flow Experience, and Self-efficacy in Creativity played key psychological roles in the development of Student Creativity. AI outputs elicited cognitive conflicts and curiosity, motivating active exploration and sustained attention while strengthening confidence in innovation and problem-solving, ultimately directly promoting creative outcomes: *“AI’s answers differed from my expectations, prompting me to investigate the reasons; when fully immersed, inspiration kept flowing; AI increased my confidence in completing innovative tasks.”*

In summary, the qualitative analysis not only corroborates the significant positive effects observed in the SEM paths but also reveals how the Exploration–Exploitation–Co-Creation–Reflection chain mechanism influences Student Creativity through cognitive stimulation, flow experience, and self-efficacy. This analysis provides micro-level psychological and behavioral explanations for the quantitative results, enriches theoretical understanding of creativity formation in AI-assisted learning, and offers empirical support for the application of generative AI in higher education (Table 8).

**Table 8:** Integration matrix of quantitative and qualitative findings.

Hypothesis	SEM Result	Coding Themes	Representative Quote	Integrated Interpretation
H1: Exploration → Perceived Cognitive Stimulation	Significant positive (β = 0.289, p < 0.001)	New Idea Generation, Knowledge Expansion, Cognitive Disruption	“AI’s answers make me reorganize my thinking.”	Students expand their knowledge boundaries and gain new cognitive stimulation through AI.
H2: Exploration → Student Creativity	Significant positive (β = 0.199, p = 0.002)	New Idea Generation, Creative Confidence	“AI helped me open up new ideas and write something novel.”	Exploratory use of AI stimulates divergent thinking and promotes creative expression.
H3: Exploitation → Perceived Cognitive Stimulation	Significant positive (β = 0.207, p = 0.003)	Structural Optimization, Efficiency Enhancement	“AI helped me organize knowledge and better understand core ideas.”	AI supports cognitive processing by helping students structure and organize knowledge.
H4: Exploitation → Student Creativity	Significant positive (β = 0.144, p = 0.04)	Efficiency Enhancement, Idea Inspiration	“Optimized content helped me generate more new ideas.”	Instrumental use of AI enhances the feasibility of creative outcomes.
H5: Co-Creation → Student Creativity	Significant positive (β = 0.185, p = 0.005)	Human–AI Collaboration, Iterative Improvement	“AI is like a collaborator; it makes my work more layered.”	Collaborative generation processes stimulate diverse creativity.
H6: Co-Creation → Flow Experience	Significant positive (β = 0.486, p < 0.001)	Human–AI Collaboration, Focused Engagement	“Repeatedly adjusting prompts makes me fully immersed.”	Co-creation interaction facilitates flow experience and learning focus.
H7: Reflection → Student Creativity	Significant positive (β = 0.119, p = 0.037)	Critical Evaluation, Learning Role Reconstruction	“I reflect on AI’s shortcomings and improve my ideas.”	Reflection promotes metacognition and creative reconstruction.
H8: Reflection → Self-efficacy in Creativity	Significant positive (β = 0.453, p < 0.001)	Learning Role Reconstruction, Creative Confidence	“AI makes me realize that creativity depends on myself.”	Reflection strengthens self-efficacy in creativity.
H9: Perceived Cognitive Stimulation → Student Creativity	Significant positive (β = 0.121, p = 0.027)	Interest Stimulation, Cognitive Disruption	“AI stimulates me to explore different solutions.”	Increased cognitive engagement promotes creative output.
H10: Flow Experience → Student Creativity	Significant positive (β = 0.116, p = 0.039)	Focused Engagement, Challenge and Control	“When fully immersed, inspiration keeps flowing.”	Flow experience translates into creative performance.
H11: Self-efficacy in Creativity → Student Creativity	Significant positive (β = 0.11, p = 0.044)	Creative Confidence, Problem-Solving Confidence	“AI gives me more confidence to complete innovative tasks.”	Self-efficacy serves as a direct psychological driver for creativity.

## 5. Discussion

### 5.1. Key research findings

Based on 424 survey responses and supplementary interviews with eight students, this study identifies a sequential Exploration–Exploitation–Co-Creation–Reflection pathway connecting GAI learning behaviors to student creativity in higher education. Quantitative results show Exploration positively predicts Student Creativity with a modest direct coefficient (β = 0.199, p = 0.002), and indirect predictive associations are observed in connection with Perceived Cognitive Stimulation (β = 0.289, p < 0.001). Exploitation similarly has a small significant direct association with creativity (β = 0.144, p = 0.04), alongside indirect predictive associations linked to cognitive stimulation (β = 0.207, p = 0.003). Interview data further illustrate that exploring new approaches and leveraging existing knowledge via GAI may help students generate alternative ideas and problem-solving thinking.

In terms of psychological mechanisms, Co-Creation displays a comparatively strong predictive link to Flow Experience (β = 0.486, p < 0.001), and Reflection positively predicts Self-efficacy in Creativity (β = 0.453, p < 0.001). However, the direct paths from flow experience and self-efficacy in creativity to Student Creativity remain weak despite statistical significance (β = 0.116, p = 0.039; β = 0.110, p = 0.044). Interview narratives echo these quantitative trends: human-AI co-creation shows positive associations with immersive learning states, while reflective practice is linked to higher confidence in students’ creative capabilities.

Taken together, the observed patterns show Exploration and Exploitation are positively associated with cognitive stimulation, while Co-Creation and Reflection are linked to motivational psychological states; these chained mechanisms collectively explain 35.9% of the variance in student creativity (R^2^ = 0.359). The results merely indicate that GAI-assisted learning behaviors correlate with changes in learners’ cognitive and motivational processes linked to creative outcomes, yet each separate behavioral dimension only holds limited unique predictive power for student creativity when considered independently.

Beyond the direct path coefficients and R^2^ values reported in Table 4, bootstrapped chain mediation tests (5,000 resamples, bias-corrected 95% CI) further examined the four hypothesized sequential indirect predictive pathways at the core of this research. The standardized indirect effect of Exploration on student creativity via Perceived Cognitive Stimulation reached 0.058, and the indirect pathway from Exploitation to creativity through cognitive stimulation was 0.041. Meanwhile, significant indirect predictive associations exist for Co-Creation in relation to student creativity in conjunction with Flow Experience (indirect effect = 0.070), and Reflection shows significant indirect predictive links to creative performance in connection with Self-efficacy in Creativity (indirect effect = 0.065). All four confidence intervals excluded zero, providing empirical support for this hypothesized chain mediation model. These formal mediation results fully support the central theoretical claim of this paper: the four-stage Exploration–Exploitation–Co-Creation–Reflection set of variables shows positive predictive associations with student creativity in connection with sequential cognitive and psychological pathways.

It is worth noting that all direct standardized paths leading to Student Creativity are relatively modest in magnitude, ranging from 0.110 to 0.199 (Self-efficacy in Creativity = 0.110, Flow Experience = 0.116, Reflection = 0.119, Perceived Cognitive Stimulation = 0.121, Exploitation = 0.144, Co-Creation = 0.185, Exploration = 0.199). Statistically significant as these links are, their relatively small individual predictive weights stem from the structural design of our multi-predictor chain mediation model. Eight interrelated behavioral, cognitive, and psychological constructs simultaneously predict student creative performance within one unified framework; substantial shared covariance across predictors inevitably reduces the unique explanatory contribution of each single path.

From an educational practice perspective, these minor single-path effects do not negate practical value. Student creativity is a cumulative outcome formed by the joint action of multiple mechanisms, rather than reflecting the influence of one single dominant factor. Although adjusting one isolated factor can only bring slight improvements to learners’ creativity, holistic interventions covering all eight constructs will generate compound and amplified gains for creative development. This delivers clear pedagogical implications: GAI creativity training should adopt integrated multi-dimensional strategies, rather than focusing on only one behavioral or psychological dimension.

### 5.2. Theoretical Explanation of the Exploration–Exploitation–Co-Creation–Reflection Mechanism

The results of the chain mediation model reveal a multi-layered mechanism associated with creativity development in GAI-assisted education. Theoretically, Exploration reflects students’ engagement in trying new approaches and identifying problems; with access to diverse materials and real-time feedback provided by GAI, such exploratory activities are associated with higher levels of Perceived Cognitive Stimulation. Exploitation reflects the application of existing knowledge and skills to specific tasks; patterns in the data show potential links between strategy-output optimization and creativity in connection with cognitive stimulation. Interview findings further suggest that students may gain not only operational experience during exploration and exploitation but also opportunities to develop innovative thinking and problem-solving abilities, indicating a possible pathway from cognitive resources to creative outcomes.

At the psychological mechanism level, Co-Creation is positively associated with Flow Experience, suggesting that collaboration with peers or GAI may show positive links to students’ task engagement and creative motivation. Reflection is positively associated with Creative Self-efficacy, indicating that reviewing and evaluating creative processes may display positive links to students’ confidence and self-regulatory capacity. Both quantitative data and interview findings consistently indicate that flow experience and self-efficacy are positively associated with creativity.

In summary, Exploration–Exploitation represents cognitive resources and methodological foundations, while Co-Creation–Reflection reflects psychological motivation and self-regulatory processes. Together, these mechanisms may constitute a systematic pathway linking behavioral engagement, psychological processes, and creative outcomes, providing insight into how GAI-assisted learning processes are associated with student creativity in higher education.

### 5.3. Mechanisms and pathways of GAI’s influence on creativity

The empirical evidence in this study identifies two interlinked, layered pathways that connect students’ GAI-assisted learning activities to creative performance, separated into cognitive and psychological dimensions. The cognitive route centers on ambidextrous Exploration–Exploitation behaviors, which show positive associations with students’ cognitive processing and divergent thinking in connection with Perceived Cognitive Stimulation. The psychological route relies on collaborative and reflective practice: Co-Creation shows positive links to immersive task states, while Reflection is associated with learners’ creative belief, with both displaying connections to stable motivational foundations for creative output.

This dual-chain framework clarifies observed patterns linking behavioral inputs to creative gains in connection with sequential cognitive and psychological progression. Interview evidence further complements this logic: when working with generative tools, students continuously iterate ideas and adjust their thinking patterns through repeated human-AI collaboration and post-task reflection, which is associated with both cognitive inspiration and internal creative motivation simultaneously.

Taken collectively, the chained sequence of “Behavior → Cognition → Psychology → Creativity” illustrates observed patterns related to GAI-supported learning processes and the developmental logic of student creativity within higher education learning contexts. This layered mechanism delivers targeted pedagogical references for designing integrated creativity training activities.

### 5.4. Integration of qualitative findings and quantitative results

This study integrates quantitative SEM analysis with qualitative interview data to develop a complementary explanatory framework. The quantitative results provide evidence regarding the significance and strength of the proposed structural relationships, while the interviews reveal students’ authentic experiences and cognitive processes during exploration, exploitation, co-creation, and reflection.

For instance, quantitative analysis indicates a significant association between exploration and perceived cognitive stimulation, which is further contextualized by students’ reports of gaining inspiration from the novel information and examples provided by GAI. Similarly, the positive relationship between co-creation and flow experience is supported by interview accounts suggesting that collaboration with peers or GAI may show positive links to task engagement and creative motivation.

The integration of qualitative and quantitative evidence not only strengthens the interpretation of the proposed model relationships but also enriches theoretical understanding by illustrating how GAI-assisted learning processes may be associated with creativity development in real educational contexts.

### 5.5. Theoretical implications and contributions

This study theoretically proposes a chain mechanism model for creativity development in GAI-assisted education, extending the application of exploration–exploitation theory to educational contexts and incorporating the co-creation–reflection psychological mechanism, thereby integrating cognitive and motivational dimensions. The study makes several theoretical contributions. First, it provides a novel perspective suggesting observed associations between GAI-assisted learning processes and creativity development in connection with multiple behavioral, cognitive, and psychological pathways. Second, it integrates behavioral, cognitive, and psychological factors into a chain model, offering empirically testable relationships for future research. Third, by combining qualitative interviews with quantitative SEM analysis, it highlights the importance of students’ experiential and cognitive processes in understanding creativity development within educational contexts. The findings also have practical implications: educators may design exploratory tasks and collaborative activities using GAI tools while encouraging reflective practices in relation to students’ creative self-efficacy. Such pedagogical approaches may display positive links to the development of students’ cognitive engagement and psychological resources in GAI-assisted learning environments.

## 6. Summary and future directions

Based on 424 survey responses and 8 interviews, this study reveals systematic predictive associations between generative artificial intelligence (GAI) and student creativity in higher education, consistent with the chain mechanism of exploration–exploitation–co-creation–reflection. The findings indicate that exploration is linked to students trying new approaches and identifying novel problems, while exploitation is associated with strengthened application of existing knowledge and skills; both pathways show indirect predictive associations with creativity in connection with perceived cognitive stimulation alongside direct predictive links. Interviews further show that students gain inspiration and strategies through practice, providing a foundation for innovative thinking. Co-creation and reflection play critical roles at the psychological level: co-creation shows positive associations with flow experience, and reflection is linked to self-efficacy in creativity, with both displaying further positive links to creative development and demonstrating the synergistic interaction between behavioral and psychological mechanisms.

Theoretically, this study integrates exploration–exploitation theory with co-creation–reflection psychological mechanisms, constructing a chain model from behavior to cognition to psychology, thereby providing a systematic framework for understanding GAI-assisted education and highlighting the central role of cognitive stimulation and motivational processes in creativity formation. Practically, the findings suggest that educators should balance exploration and exploitation when designing GAI-assisted learning activities, complemented by collaborative co-creation and reflective practices to comprehensively foster student creativity.

Nonetheless, the study still bears several critical limitations. First, this research adopts a single-wave, cross-sectional single-source self-report design. As such, we can only interpret correlational and predictive associations among variables, and definitive causal conclusions cannot be drawn; reverse causality cannot be fully ruled out. Additionally, we did not recruit a comparison group of students who rarely or never use generative AI, which means we are unable to fully isolate the unique predictive effect of GAI engagement on creativity. All participants were recruited from a single university, further restricting cross-institutional generalizability. Regarding the qualitative strand, all eight interviewees were active GAI users. Although thematic saturation was fully achieved to capture core themes of the proposed chain mechanism, no strong disconfirming cases that contradict the model emerged from this user-only sample—only mixed and ambivalent experiential accounts were identified. The small qualitative sample lacking non-user perspectives limits broad cross-group extrapolation of interview insights.

Second, the model exhibits limited explained variance for the mediating latent variables (R² = 0.196–0.236). The current research framework only incorporates four behavioral predictors (Exploration, Exploitation, Co-Creation, Reflection) to predict cognitive and psychological mediators, yet a wide range of critical antecedents of student creativity are excluded from the model, including personality traits, disciplinary differences, teacher instructional support, length of GAI usage, and peer interaction quality. These omitted exogenous variables can account for the substantial unexplained variance of the mediators and creative outcome.

Third, the current research does not include a dedicated latent construct to capture the intensity, depth and quality of students’ GAI tool engagement. Data about the duration and types of GAI usage are only collected as background descriptive information and are not incorporated into the structural model. Therefore, this model cannot assess whether differences in technology-use characteristics moderate or predict the chain mediation effects observed in this study. This work centers on learners’ cognitive and behavioral patterns when learning with generative AI, rather than disentangling the independent influence of technology usage characteristics on creativity.

Furthermore, we only tested the theoretically hypothesised chain mediation model without designing and fitting plausible alternative competing structural models for comparative fit evaluation, which limits the empirical evidence supporting the uniqueness of the proposed sequential pathway structure. Future research can construct multiple nested alternative models and compare their fit indices to further verify the structural rationality of this Exploration–Exploitation–Co-Creation–Reflection framework.

Future research may also adopt longitudinal multi-wave designs and recruit non-GAI user comparison groups to enable more rigorous causal inference; it could also expand qualitative samples to include non-users to capture divergent viewpoints. Researchers may further develop targeted scales measuring GAI engagement quality and introduce these indicators as predictors or moderators to advance understanding of AI-supported creative learning, while integrating omitted individual and contextual antecedents into the analytical framework. Overall, this study not only elucidates multi-pathway patterns of association between GAI and creativity in higher education but also offers theoretical and practical insights for the deep integration of artificial intelligence into education.

## Supporting information

S1 File Anonymized quantitative dataset. Raw quantitative research data.
(XLSX)

S2 File Qualitative coding materials. Audio-to-text transcribed documents for qualitative analysis.
(DOCX)

S3 File Survey and interview protocols. Appendix A measurement items and Appendix B interview questions used in this study.
(DOCX)

## References

[1] Habib S, Vogel T, Anli X, Thorne E. How does generative artificial intelligence impact student creativity?. Journal of Creativity. 2024;34(1):100072. doi: 10.1016/j.yjoc.2023.100072

[2] Chan CKY, Hu W. Students’ voices on generative AI: perceptions, benefits, and challenges in higher education. Int J Educ Technol High Educ. 2023;20(1). doi: 10.1186/s41239-023-00411-8

[3] Kasneci E, Sessler K, Küchemann S, Bannert M, Dementieva D, Fischer F, et al. ChatGPT for good? On opportunities and challenges of large language models for education. Learning and Individual Differences. 2023;103:102274. doi: 10.1016/j.lindif.2023.102274

[4] Amabile TM. Creativity in context: Update to the social psychology of creativity. Routledge. 2018.

[5] Csikszentmihalyi M, Csikzentmihaly M. Flow: The psychology of optimal experience. New York: Harper & Row. 1990.

[6] Holmes W, Bialik M, Fadel C. Artificial intelligence in education promises and implications for teaching and learning. Center for Curriculum Redesign. 2019.

[7] Tian Z, Wang C. Rethinking Language Education in the Age of Generative AI. Taylor & Francis Group. 2025.

[8] Tlili A, Shehata B, Adarkwah MA, Bozkurt A, Hickey DT, Huang R, et al. What if the devil is my guardian angel: ChatGPT as a case study of using chatbots in education. Smart Learn Environ. 2023;10(1). doi: 10.1186/s40561-023-00237-x

[9] Zhai X. ChatGPT user experience: Implications for education. 2022. https://ssrn.com/abstract=4312418

[10] March JG. Exploration and Exploitation in Organizational Learning. Organization Science. 1991;2(1):71–87. doi: 10.1287/orsc.2.1.71

[11] Zhou Q, Dekkers R, Chia R. Are James March’s ‘exploration’ and ‘exploitation’ separable? Revisiting the dichotomy in the context of innovation management. Technological Forecasting and Social Change. 2023;192:122592. doi: 10.1016/j.techfore.2023.122592

[12] Gupta AK, Smith KG, Shalley CE. The interplay between exploration and exploitation. AMJ. 2006;49(4):693–706. doi: 10.5465/amj.2006.22083026

[13] Riedl MO. Human‐centered artificial intelligence and machine learning. Hum Behav & Emerg Tech. 2019;1(1):33–6. doi: 10.1002/hbe2.117

[14] Luckin R, Holmes W. Intelligence unleashed: An argument for AI in education. 2016.

[15] Roll I, Winne PH. Understanding, evaluating, and supporting self-regulated learning using learning analytics. Learning Analytics. 2015;2(1):7–12. doi: 10.18608/jla.2015.21.2

[16] Dwivedi YK, Kshetri N, Hughes L, Slade EL, Jeyaraj A, Kar AK, et al. Opinion Paper: “So what if ChatGPT wrote it?” Multidisciplinary perspectives on opportunities, challenges and implications of generative conversational AI for research, practice and policy. Int J Inform Manag. 2023;71:102642.

[17] Xu X, Qiao L, Cheng N, Liu H, Zhao W. Enhancing self‐regulated learning and learning experience in generative AI environments: The critical role of metacognitive support. Brit J Educational Tech. 2025;56(5):1842–63. doi: 10.1111/bjet.13599

[18] Cropley A. In praise of convergent thinking. Creativity Research J. 2006;18(3):391–404. doi: 10.1207/s15326934crj1803_13

[19] Lee D, Palmer E. Prompt engineering in higher education: A systematic review to help inform curricula. Int J Educ Technol High Educ. 2025;22(1). doi: 10.1186/s41239-025-00503-7

[20] Corbeil JR, Corbeil ME. Teaching and Learning in the Age of Generative AI: Evidence-based Approaches to Pedagogy, Ethics, and Beyond. Taylor & Francis. 2025.

[21] Shi SJ, Li JW, Zhang R. A study on the impact of Generative Artificial Intelligence supported Situational Interactive Teaching on students’ ‘flow’ experience and learning effectiveness — a case study of legal education in China. Asia Pacific Journal of Education. 2024;44(1):112–38. doi: 10.1080/02188791.2024.2305161

[22] Bandura A. Self-efficacy: The exercise of control. Macmillan. 1997.

[23] Karwowski M, Lebuda I, Wisniewska E, Gralewski J. Big Five Personality Traits as the Predictors of Creative Self‐Efficacy and Creative Personal Identity: Does Gender Matter?. Journal of Creative Behavior. 2013;47(3):215–32. doi: 10.1002/jocb.32

[24] Cubillos C, Mellado R, Cabrera-Paniagua D, Urra E. Generative Artificial Intelligence in Computer Programming: Does it enhance learning, motivation, and the learning environment?. IEEE Access. 2025.

[25] McGrath C, Farazouli A, Cerratto-Pargman T. Generative AI chatbots in higher education: a review of an emerging research area. High Educ. 2024;89(6):1533–49. doi: 10.1007/s10734-024-01288-w

[26] Andriopoulos C, Lewis MW. Exploitation-exploration tensions and organizational ambidexterity: managing paradoxes of innovation. Organization Sci. 2009;20(4):696–717. doi: 10.1287/orsc.1080.0406

[27] Zhou J, George JM. When job dissatisfaction leads to creativity: encouraging the expression of voice. Academy of Management J. 2001;44(4):682–96. doi: 10.2307/3069410

[28] Wei X, Wang L, Lee L-K, Liu R. The effects of generative AI on collaborative problem-solving and team creativity performance in digital story creation: an experimental study. Int J Educ Technol High Educ. 2025;22(1). doi: 10.1186/s41239-025-00526-0

[29] Tierney P, Farmer SM. Creative self-efficacy: its potential antecedents and relationship to creative performance. Acad Manag J. 2002;45(6):1137–48. doi: 10.2307/3069429

[30] Beghetto RA, Kaufman JC. Classroom contexts for creativity. High Ability Studies. 2014;25(1):53–69. doi: 10.1080/13598139.2014.905247

[31] Qian Y. Pedagogical applications of generative AI in higher education: A systematic review of the field. TechTrends. 2025;69(5):1105–20. doi: 10.1007/s11528-025-01100-1

[32] Fong CJ, Zaleski DJ, Leach JK. The challenge–skill balance and antecedents of flow: A meta-analytic investigation. J Positive Psychol. 2014;10(5):425–46. doi: 10.1080/17439760.2014.967799

[33] Phan HP. Exploring students’ reflective thinking practice, deep processing strategies, effort, and achievement goal orientations. Educat Psychol. 2009;29(3):297–313. doi: 10.1080/01443410902877988

[34] Sun JC, Rueda R. Situational interest, computer self‐efficacy and self‐regulation: Their impact on student engagement in distance education. Brit J Educat Tech. 2011;43(2):191–204. doi: 10.1111/j.1467-8535.2010.01157.x

[35] Kaufman JC. Creativity 101. Springer Publishing Company. 2016.

[36] Hwang Y, Lee JH. Exploring students’ experiences and perceptions of human-AI collaboration in digital content making. Int J Educ Technol High Educ. 2025;22(1). doi: 10.1186/s41239-025-00542-0

[37] McGuire J, De Cremer D, Van de Cruys T. Establishing the importance of co-creation and self-efficacy in creative collaboration with artificial intelligence. Sci Rep. 2024;14(1):18525. doi: 10.1038/s41598-024-69423-2 39122865 PMC11316096

[38] Beghetto R. Creative self-efficacy: Correlates in middle and secondary students. Creativity Res J. 2006;18(4):447–57. doi: 10.1207/s15326934crj1804_4

[39] Hair Jr JF, Black WC, Babin BJ, Anderson RE. Multivariate data analysis. Multivariate data analysis. 2010. p. 785.

[40] Barrett P. Structural equation modelling: Adjudging model fit. Personality and Individual Differences. 2007;42(5):815–24. doi: 10.1016/j.paid.2006.09.018

[41] Podsakoff PM, MacKenzie SB, Lee J-Y, Podsakoff NP. Common method biases in behavioral research: a critical review of the literature and recommended remedies. J Appl Psychol. 2003;88(5):879–903. doi: 10.1037/0021-9010.88.5.879 14516251

[42] Shiau W-L, Sarstedt M, Hair JF. Internet research using partial least squares structural equation modeling (PLS-SEM). INTR. 2019;29(3):398–406. doi: 10.1108/intr-10-2018-0447

[43] Fornell C, Larcker DF. Evaluating Structural Equation Models with Unobservable Variables and Measurement Error. J Marketing Res. 1981;18(1):39. doi: 10.2307/3151312

[44] Henseler J, Ringle CM, Sarstedt M. A new criterion for assessing discriminant validity in variance-based structural equation modeling. J Acad Mark Sci. 2014;43(1):115–35. doi: 10.1007/s11747-014-0403-8

[45] Hair JF Jr, Babin BJ, Krey N. Covariance-based structural equation modeling in the journal of advertising: review and recommendations. J Advertising. 2017;46(3):454–454. doi: 10.1080/00913367.2017.1329496

[46] Hu L, Bentler PM. Fit indices in covariance structure modeling: Sensitivity to underparameterized model misspecification. Psychol Methods. 1998;3(4):424–53. doi: 10.1037/1082-989x.3.4.424

[47] Bagozzi RP, Yi Y. On the evaluation of structural equation models. JAMS. 1988;16(1):74–94. doi: 10.1007/bf02723327

[48] Hayduk LA. Structural equation modeling with LISREL: Essentials and advances. Jhu Press. 1987.

[49] Scott JE. The measurement of information systems effectiveness: evaluating a measuring instrument. ACM SIGMIS Database. 1995;26(1):43–61.

[50] Hayes AF, Little TD. Introduction to mediation, moderation, and conditional process analysis: regression-based approach. Second ed. The Guilford Press. 2018.

[51] Preacher KJ, Hayes AF. Asymptotic and resampling strategies for assessing and comparing indirect effects in multiple mediator models. Behav Res Methods. 2008;40(3):879–91. doi: 10.3758/brm.40.3.879 18697684

[52] Braun V, Clarke V. Using thematic analysis in psychology. Qualitative Res Psychol. 2006;3(2):77–101. doi: 10.1191/1478088706qp063oa

