# Supplementary material for: How Generative Artificial Intelligence (GAI) reshapes students’ creativity in higher education: A chain path modeling Study from “Exploration–Exploitation” to “Co-Creation–Reflection”
Source: PLoS One. 2026 Sep 24;21(9):e0358186. doi: 10.1371/journal.pone.0358186 (PMC13606950; doi:10.1371/journal.pone.0358186)
Supplement: Supplementary file 1 [file pone.0358186.s002.docx]

**Participant: P01**

Interviewer: When using AI to complete learning tasks, do you try different approaches or new methods?

P01: Yes, I do. AI allows me to try different ways of expressing ideas and different ways of thinking during the learning process. For example, when writing a paper, I sometimes ask AI to help me analyze the same issue from different theoretical perspectives.

Interviewer: Do you use AI to explore knowledge or viewpoints that you were previously unfamiliar with?

P01: Yes. I often use AI to learn about interdisciplinary topics. For example, when studying educational theories, I ask AI to explain concepts related to psychology or sociology. This helps me broaden my perspective and think beyond my original understanding.

Interviewer: Could you give an example of how AI has helped you “open up your thinking”?

P01:When I was writing a course paper, my original research perspective was quite limited. Later, AI suggested that I look at the issue by combining two variables: “learning motivation” and “digital technology adaptability.” This made me realize that I could build a research framework from a more multidimensional perspective.

Interviewer: How is this experience of “exploration” different from your previous ways of learning?

P01:In the past, my learning mainly depended on textbooks and guidance from teachers. With AI, I can ask questions on my own and receive feedback immediately. This interactive exploration feels much stronger, and it also motivates me to take a more active role in my learning.

Interviewer: Do you use AI to improve or refine your existing learning outcomes?

P01:Yes, I do. I often use AI to help me improve the logical structure and language expression of my papers, which helps enhance the overall quality of my work.

Interviewer: When using AI, do you tend to let it generate new content or help you optimize your existing ideas?

P01:I prefer using it to improve my existing ideas. Although generating new content is convenient, I think it is more important to develop deeper insights and make my ideas clearer and more coherent based on what I already have.

Interviewer: Has AI helped you improve your learning efficiency or the quality of your work? In what way?

P01:Yes, it has improved them significantly. AI can quickly help me find information and summarize literature, which saves me a lot of time. It also allows me to focus more on the analysis and critical thinking parts of my work.

Interviewer: How do you decide whether the information provided by AI is reliable or useful?

P01:I usually compare it with academic databases or textbooks for verification. Especially when dealing with professional topics, I check the original studies or peer-reviewed sources to make sure the information is accurate and reliable.

Interviewer: When using AI, do you feel that it stimulates your thinking or learning interest?

P01:Yes, I do. AI often raises questions or provides examples that I have not considered before, which encourages me to explore the topic further.

Interviewer: How do you respond when AI provides answers that differ from your expectations?

P01:I do not immediately reject it. Instead, I try to understand why AI gave that response and reflect on whether my own understanding might be incomplete or one-sided.

Interviewer: Do these unexpected results make you think more deeply about the problem?

P01:Yes, they do. In many cases, AI’s “mistakes” can actually inspire me to reconsider the problem itself from a different perspective.

Interviewer: Have you discovered new perspectives or connections as a result?

P01:Yes. For example, when discussing “creativity,” AI helped me realize that creativity is not only an individual ability but is also influenced by social and cultural contexts. This helped me develop a deeper understanding of the concept.

Interviewer: Do you feel particularly focused or immersed when using AI for learning?

P01:Yes, I do. Especially when I keep asking follow-up questions and refining my prompts with AI, I often enter a state of deep focus and concentration.

Interviewer: How does the learning experience with AI differ from traditional learning methods?

P01:AI provides more immediate and personalized feedback. I can learn at my own pace and adjust the learning process according to my needs. This level of autonomy is difficult to achieve in traditional classroom settings.

Interviewer: When AI’s response is unsatisfactory, do you give up or try to adjust it?

P01:I usually try to adjust the way I ask questions. In most cases, if I approach the question from a different angle or provide more background information, AI’s responses become more relevant to what I need.

Interviewer: Does this experience make your learning feel easier or more challenging?

P01:I think it is both. It makes learning easier because I can access resources more quickly, but it also brings challenges because I need to have critical judgment skills to evaluate the quality of AI-generated information.

Interviewer: After using AI, do you feel more confident in proposing creative ideas or solving problems?

P01:Yes, I do. AI allows me to quickly test the feasibility of my ideas, which increases my confidence in developing creative solutions.

Interviewer: Do you think AI has helped you improve your learning ability?

P01:Definitely. It has especially improved my abilities in information integration and logical expression because I need to evaluate, select, and reorganize the content generated by AI.

Interviewer: Do you think AI makes you more willing to try new methods? Why?

P01:Yes. Since AI can provide immediate feedback and multiple possible approaches, I feel that the risks of trying new methods are lower, and I have more confidence to experiment.

Interviewer: Can you recall an experience with AI that made you feel, “I can actually do this”?

P01:Yes. Once, when I was writing a research report, AI helped me organize the analytical framework. Based on that, I further improved my model design, and my supervisor gave me positive feedback. That experience made me realize that my research ability was improving.

Interviewer: When using AI, do you consider it a learning partner or collaborator? Why or why not?

P01:Yes, I do. Although AI is not human, its feedback can be inspiring and interactive. In the learning process, it feels more like a “mirror for my thinking” that helps me reflect on and develop my ideas.

Interviewer: Through interacting with AI, have you rethought your learning methods or ideas?

P01:Yes. I realized that I used to rely too much on linear thinking, while AI encourages me to understand problems in a more systematic and divergent way.

Interviewer: Do you modify or refine your ideas with AI’s help?

P01:I do this quite often. Especially during the writing and research design stages, AI’s suggestions often help me identify logical gaps or unclear expressions in my work.

Interviewer: Do you reflect on AI’s limitations and your own role in the learning process?

P01:Yes. Although AI is efficient, it lacks value judgment and genuine creative thinking. I believe that humans should remain the decision-makers, while AI should serve as a supporting tool.

Interviewer: Do you think using AI makes your learning outcomes more creative or of higher quality?

P01:AI allows me to integrate different perspectives within a shorter period of time, which helps improve the depth and creativity of my work.

Interviewer: Could you give an example of how AI has helped you improve your results?

P01:In a course project, I used AI to analyze user feedback data. It helped me identify some potential variables, which made my report more insightful and comprehensive.

Interviewer: Without AI, do you think you could have achieved similar learning outcomes?

P01:It would be difficult. AI has greatly improved my efficiency and expanded the range of information I can access. Without it, I would probably need much more time to complete the same tasks.

Interviewer: Do you think AI’s greatest contribution lies in “inspiring thinking” or “completing tasks”?

P01:I think it mainly helps with “inspiring thinking.” AI encourages me to keep asking questions, refining my ideas, and reflecting on my understanding. I think this is the most valuable part of the learning process.

**Participant: P02**

Interviewer: When using AI to complete learning tasks, do you try different approaches or new methods?

P02: Yes, I do. AI’s responses often inspire me to explore directions that I would not have considered before, such as using different styles or structures to present design concepts. I enjoy continuously modifying my prompts and observing the changes. The process itself feels like exploring new ways of thinking.

Interviewer: Do you use AI to explore knowledge or viewpoints that you were previously unfamiliar with?

P02: Yes. I often ask AI to explain design theories or art movements that I do not fully understand, such as Constructivism or the Bauhaus style. AI helps me quickly understand the background and core ideas, and then I combine this with visual examples for deeper learning.

Interviewer: Could you give an example of how AI has helped you “open up your thinking”?

P02:Once, when I was working on a brand visual design project, AI suggested that I approach the graphic language from the perspective of brand emotions rather than starting from product functions. This completely changed my way of thinking and made the work more emotionally engaging.

Interviewer: How is this experience of “exploration” different from your previous ways of learning?

P02:Previously, my learning relied more on teachers and textbooks, so the process was relatively linear. The interaction with AI allows me to immediately try different perspectives, making my thinking more open, flexible, and proactive.

Interviewer: Do you use AI to improve or refine your existing learning outcomes?

P02:Yes, I do. For example, in design projects, I use AI to help me analyze whether the layout is balanced, whether the text is too repetitive, or to polish my project descriptions so that my ideas can be expressed more clearly.

Interviewer: When using AI, do you tend to let it generate new content or help you optimize your existing ideas?

P02:At first, I relied more on AI to generate new content, but now I prefer using it to improve my existing ideas because this helps maintain my personal style in my work.

Interviewer: Has AI helped you improve your learning efficiency or the quality of your work? In what way?

P02:Yes, it has improved them a lot. AI can quickly provide multiple reference options, which helps me decide on a direction faster. It also saves me a significant amount of time searching for information and making layout adjustments.

Interviewer: How do you decide whether the information provided by AI is reliable or useful?

P02:I usually check the sources or compare answers from different AI tools. If it involves theoretical content, I will also refer to academic literature or textbooks for confirmation.

Interviewer: When using AI, do you feel that it stimulates your thinking or learning interest?

P02:Yes, I do. AI’s responses often surprise me, and they stimulate my curiosity to ask further questions and verify the information.

Interviewer: How do you respond when AI provides answers that differ from your expectations?

P02:I do not reject it immediately. Instead, I try to understand why it responded that way. Sometimes I realize that the problem was actually caused by the way I asked the question being unclear.

Interviewer: Do these unexpected results make you think more deeply about the problem?

P02:Yes. I will reorganize the logic of the problem and sometimes even redefine the objectives of the task.

Interviewer: Have you discovered new perspectives or connections as a result?

P02:Yes, quite often. For example, when AI connected “spatial design” with “emotional expression,” I realized that visual design is not only about form but also a way of communicating psychological experiences.

Interviewer: Do you feel particularly focused or immersed when using AI for learning?

P02:Yes, I do. When I keep adjusting prompts or trying to generate different styles, I often become so engaged that I lose track of time.

Interviewer: How does the learning experience with AI differ from traditional learning methods?

P02:AI provides immediate feedback and strong interaction. I can instantly see visualized results of my ideas, while traditional learning methods are often slower and more linear.

Interviewer: When AI’s response is unsatisfactory, do you give up or try to adjust it?

P02:I usually try to make adjustments, such as changing the way I describe my ideas or providing more detailed requirements.

Interviewer: Does this experience make your learning feel easier or more challenging?

P02:I think it is both. It makes learning easier because I can access information more quickly, but creating satisfactory outcomes still requires continuous trial and adjustment, which makes the process challenging.

Interviewer: After using AI, do you feel more confident in proposing creative ideas or solving problems?

P02:Yes, I do. AI shows me that ideas can be expanded in unlimited ways, and it has helped me build more confidence in developing creative ideas.

Interviewer: Do you think AI has helped you improve your learning ability?

P02:Yes, it has helped me a lot. Through interacting with AI, I have learned how to ask questions and express my ideas in a more systematic way, and I can also learn new tools more quickly.

Interviewer: Do you think AI makes you more willing to try new methods? Why?

P02:Yes. AI provides a safe “experimental space.” Even if an attempt does not work well, I can try again and continue improving without feeling pressured.

Interviewer: Can you recall an experience with AI that made you feel, “I can actually do this”?

P02:Yes. I once used Midjourney to generate a series of visual concepts. My supervisor commented that my creative perspective had become more mature. At that moment, I felt that AI helped me achieve a level of creative expression beyond what I had been able to do before.

Interviewer: When using AI, do you consider it a learning partner or collaborator? Why or why not?

P02:Yes, I do. I see it more as an “intelligent conversational partner.” It does not create for me, but instead helps me organize my thoughts and reflect on my ideas.

Interviewer: Through interacting with AI, have you rethought your learning methods or ideas?

P02:Yes. I realized that learning is not only about obtaining answers; more importantly, it is about how to ask questions and reflect on the process.

Interviewer: Do you modify or refine your ideas with AI’s help?

P02:I do this quite often. AI provides suggestions from different perspectives, and I select the valuable parts and integrate them into my work.

Interviewer: Do you reflect on AI’s limitations and your own role in the learning process?

P02:Yes. I understand that AI’s creativity is based on existing data rather than true originality. Therefore, I still need to maintain independent thinking and my own aesthetic judgment.

Interviewer: Do you think using AI makes your learning outcomes more creative or of higher quality?

P02:Yes, I do. The overall quality of my work has improved, and my design expression has also become deeper and more meaningful.

Interviewer: Could you give an example of how AI has helped you improve your results?

P02:In a poster design project, AI helped me reorganize the visual hierarchy, making the main theme more prominent and improving the rhythm and balance of the layout.

Interviewer: Without AI, do you think you could have achieved similar learning outcomes?

P02:It would be difficult. The efficiency and expanded perspectives provided by AI allow me to generate more design options within a shorter period of time.

Interviewer: Do you think AI’s greatest contribution lies in “inspiring thinking” or “completing tasks”?

P02:For me, it is mainly “inspiring thinking.” It has changed the way I view creativity and design, and it has taught me to approach innovation with a more systematic way of thinking.

**Participant: P03**

Interviewer: When using AI to complete learning tasks, do you try different approaches or new methods?

P03: Yes, I do. For example, when reviewing literature or designing experiments, I ask AI to analyze issues from different perspectives. Sometimes it even inspires me to explore new research directions.

Interviewer: Do you use AI to explore knowledge or viewpoints that you were previously unfamiliar with?

P03: Yes. I also use AI to explore unfamiliar areas, such as the applications of machine learning in medical diagnosis. AI can explain complex models in a more understandable way, which helps me quickly build a basic understanding.

Interviewer: Could you give an example of how AI has helped you “open up your thinking”?

P03:Once, I asked AI to help me analyze the mechanism of action of a certain drug. It introduced a signaling pathway that I had not considered before, which made me rethink my research direction.

Interviewer: How is this experience of “exploration” different from your previous ways of learning?

P03:Compared with simply relying on textbooks in the past, this exploration experience is more flexible and open. Previously, my learning was more passive, but now I can actively propose hypotheses and receive feedback immediately.

Interviewer: Do you use AI to improve or refine your existing learning outcomes?

P03:I often use AI to improve the structure of my papers, optimize academic expression, or add details to my experimental designs.

Interviewer: When using AI, do you tend to let it generate new content or help you optimize your existing ideas?

P03:Compared with generating completely new content, I prefer using AI to optimize my existing ideas or drafts.

Interviewer: Has AI helped you improve your learning efficiency or the quality of your work? In what way?

P03:Yes, it has. For example, when writing literature reviews, AI can help me organize the logic more quickly and reduce repetitive work.

Interviewer: How do you decide whether the information provided by AI is reliable or useful?

P03:I usually verify the information through cross-checking and reviewing original literature. Especially in medical research, I pay close attention to the accuracy of data sources.

Interviewer: When using AI, do you feel that it stimulates your thinking or learning interest?

P03:Yes, it does. Some of AI’s unexpected or counterintuitive responses often raise new questions and encourage me to explore further.

Interviewer: How do you respond when AI provides answers that differ from your expectations?

P03:When that happens, I usually ask “why” and try to understand the logic behind its response.

Interviewer: Do these unexpected results make you think more deeply about the problem?

P03:Yes. These unexpected ideas often encourage me to think more deeply rather than simply accept the answer directly.

Interviewer: Have you discovered new perspectives or connections as a result?

P03:Yes. Sometimes AI helps me discover connections between different types of knowledge, such as combining clinical phenomena with molecular mechanisms to analyze problems from a new perspective.

Interviewer: Do you feel particularly focused or immersed when using AI for learning?

P03:Yes. I am often highly focused when using AI for learning, especially when dealing with complex analysis or writing tasks. Sometimes I can enter a state of “flow.”

Interviewer: How does the learning experience with AI differ from traditional learning methods?

P03:Compared with traditional learning, AI is more interactive. I can receive feedback immediately instead of waiting for guidance from teachers.

Interviewer: When AI’s response is unsatisfactory, do you give up or try to adjust it?

P03:I usually adjust the way I ask questions rather than giving up.

Interviewer: Does this experience make your learning feel easier or more challenging?

P03:I think it makes learning more challenging but also more interesting. It saves time while encouraging deeper thinking.

Interviewer: After using AI, do you feel more confident in proposing creative ideas or solving problems?

P03:Yes. AI has increased my confidence in developing innovative ideas because it allows me to see different ways of thinking from multiple perspectives.

Interviewer: Do you think AI has helped you improve your learning ability?

P03:Yes, I think AI has improved my learning abilities, especially in terms of logical integration and academic expression.

Interviewer: Do you think AI makes you more willing to try new methods? Why?

P03:Yes. AI encourages me to try new methods because the cost of failure becomes lower, and I can adjust my ideas whenever needed.

Interviewer: Can you recall an experience with AI that made you feel, “I can actually do this”?

P03:Yes. Once, I used AI to help me write a research abstract. My supervisor commented that the ideas were very innovative, and that made me realize, “I can actually achieve this kind of creative expression.”

Interviewer: When using AI, do you consider it a learning partner or collaborator? Why or why not?

P03:Yes. I think AI is like a learning partner rather than just a tool. It can communicate with me and help me collaboratively improve my ideas.

Interviewer: Through interacting with AI, have you rethought your learning methods or ideas?

P03:Yes. Through interacting with AI, I have reconsidered my learning approach, such as shifting from passively receiving information to actively asking questions.

Interviewer: Do you modify or refine your ideas with AI’s help?

P03:Yes, I often revise my research hypotheses or writing structures based on AI’s feedback.

Interviewer: Do you reflect on AI’s limitations and your own role in the learning process?

P03:Yes. I also reflect on AI’s limitations, such as its ability to sometimes generate inaccurate information. Therefore, I realize that I must maintain critical thinking. AI should be a supporting tool rather than a replacement.

Interviewer: Do you think using AI makes your learning outcomes more creative or of higher quality?

P03:Yes, I do. AI has improved the innovation and quality of my learning outcomes, especially in terms of research ideas and the structure of academic expression.

Interviewer: Could you give an example of how AI has helped you improve your results?

P03:For example, when preparing a review article on early cancer screening, AI helped me integrate perspectives from different fields, making the content more creative and comprehensive.

Interviewer: Without AI, do you think you could have achieved similar learning outcomes?

P03:I think I could still complete the work without AI, but it would not be as efficient or systematic, especially in terms of integrating information.

Interviewer: Do you think AI’s greatest contribution lies in “inspiring thinking” or “completing tasks”?

P03:I think AI’s greatest contribution is “inspiring thinking.” It helps me reconsider problems from different perspectives rather than simply helping me complete tasks.

**Participant: P04**

Interviewer: When using AI to complete learning tasks, do you try different approaches or new methods?

P04: Yes, I do. For example, when analyzing social policies, I ask AI to explain issues from different perspectives, such as economic, cultural, and gender perspectives.

Interviewer: Do you use AI to explore knowledge or viewpoints that you were previously unfamiliar with?

P04: Yes. I often use AI to learn about unfamiliar fields, such as computational social science and statistical modeling, which helps broaden my research perspectives.

Interviewer: Could you give an example of how AI has helped you “open up your thinking”?

P04:For example, when I was researching social inequality issues, AI reminded me to consider the perspective of “intergenerational mobility,” which inspired me to develop a new research hypothesis.

Interviewer: How is this experience of “exploration” different from your previous ways of learning?

P04:This exploration experience is more open and creative than before. Previously, I mainly relied on literature, but now I can generate different ideas immediately and compare them with each other.

Interviewer: Do you use AI to improve or refine your existing learning outcomes?

P04:I often use AI to revise the structure of my papers or improve the logic of my existing arguments.

Interviewer: When using AI, do you tend to let it generate new content or help you optimize your existing ideas?

P04:Compared with generating entirely new content, I prefer using AI to optimize my existing ideas, such as improving the structure of my arguments or expressing my viewpoints more clearly.

Interviewer: Has AI helped you improve your learning efficiency or the quality of your work? In what way?

P04:Yes. AI has improved my writing efficiency, especially when organizing literature and developing theoretical frameworks. It can help me quickly identify themes and keywords.

Interviewer: How do you decide whether the information provided by AI is reliable or useful?

P04:When evaluating the reliability of information, I usually ask AI to provide source links or references and then verify them through academic databases.

Interviewer: When using AI, do you feel that it stimulates your thinking or learning interest?

P04:Yes, I think AI can stimulate my thinking, especially when it provides answers that differ from my expectations. It encourages me to reflect on my own assumptions.

Interviewer: How do you respond when AI provides answers that differ from your expectations?

P04:I ask further questions about “why” and try to understand the theoretical logic behind the differences.

Interviewer: Do these unexpected results make you think more deeply about the problem?

P04:Yes. These unexpected feedback often lead me to analyze problems more deeply and sometimes even inspire new research questions.

Interviewer: Have you discovered new perspectives or connections as a result?

P04:Yes. With AI’s inspiration, I have gradually learned to examine social phenomena from multiple dimensions, such as combining institutional factors with individual behaviors.

Interviewer: Do you feel particularly focused or immersed when using AI for learning?

P04:Yes. I often become highly engaged when using AI, especially during data analysis or theoretical writing, and sometimes I enter a state where I become completely absorbed in the task.

Interviewer: How does the learning experience with AI differ from traditional learning methods?

P04:It feels like communicating with a research assistant that is available for discussion at any time.

Interviewer: When AI’s response is unsatisfactory, do you give up or try to adjust it?

P04:I usually adjust my questions or keywords. Often, improving the way I communicate with AI leads to better responses.

Interviewer: Does this experience make your learning feel easier or more challenging?

P04:I think it is both. AI makes information access easier, but it also requires me to evaluate, question, and improve my own ideas.

Interviewer: After using AI, do you feel more confident in proposing creative ideas or solving problems?

P04:Yes. AI helps me examine whether my ideas are logically reasonable, which gives me more confidence to develop new perspectives.

Interviewer: Do you think AI has helped you improve your learning ability?

P04:Yes. Beyond improving the final outcome, AI has helped me develop clearer thinking patterns and improve the way I construct arguments.

Interviewer: Do you think AI makes you more willing to try new methods? Why?

P04:Yes. Since AI can provide basic explanations of unfamiliar theories or methods, I feel more willing to explore interdisciplinary approaches.

Interviewer: Can you recall an experience with AI that made you feel, “I can actually do this”?

P04:Yes. I used AI to develop an interview guide for my research. The questions it suggested had a deeper structure than my initial version, which made me realize that I could also design more sophisticated research materials.

Interviewer: When using AI, do you consider it a learning partner or collaborator? Why or why not?

P04:Yes. I see AI as a research collaborator that helps transform initial ideas into clearer and more structured arguments.

Interviewer: Through interacting with AI, have you rethought your learning methods or ideas?

P04:Yes. It made me realize that learning is not only about collecting information but also about continuously questioning and reorganizing knowledge.

Interviewer: Do you modify or refine your ideas with AI’s help?

P04:Yes. I often revise my research structure or expand my analytical perspectives after discussing ideas with AI.

Interviewer: Do you reflect on AI’s limitations and your own role in the learning process?

P04:Yes. AI may not fully understand complex social contexts, so human researchers are still essential for interpreting meanings and social realities.

Interviewer: Do you think using AI makes your learning outcomes more creative or of higher quality?

P04:Yes. It has improved my academic expression and helped me build stronger logical structures in my research.

Interviewer: Could you give an example of how AI has helped you improve your results?

P04:When writing a paper about the digital divide, AI helped me connect ideas from different international studies, which allowed me to develop a broader research perspective.

Interviewer: Without AI, do you think you could have achieved similar learning outcomes?

P04:I could complete the work, but the process would be slower and less flexible. AI allows me to continuously adjust and improve my research direction.

Interviewer: Do you think AI’s greatest contribution lies in “inspiring thinking” or “completing tasks”?

P04:For me, it is inspiring thinking. AI works like a mirror that helps me recognize the limits of my own knowledge and rethink how I approach research problems.

**Participant: P05**

Interviewer: When using AI to complete learning tasks, do you try different approaches or new methods?

P05: Yes. When using AI for composition, I continuously try different prompts or styles to let AI generate different musical structures or harmonic progression options.

Interviewer: Do you use AI to explore knowledge or viewpoints that you were previously unfamiliar with?

P05: Yes. I also use AI to explore some composition techniques that I was not familiar with before, such as electronic music programming and the logic behind AI-generated melodies.

Interviewer: Could you give an example of how AI has helped you “open up your thinking”?

P05:For example, when I was creating a piece with a Chinese folk music theme, AI suggested combining Chinese traditional musical modes with jazz harmony. It generated an unexpected musical passage, which helped me find a completely new direction for expression.

Interviewer: How is this experience of “exploration” different from your previous ways of learning?

P05:This exploration experience is completely different from before. Previously, I relied more on intuition, but with AI’s involvement, I can systematically try different composition approaches, making the process more experimental and open.

Interviewer: Do you use AI to improve or refine your existing learning outcomes?

P05:Yes. I often use AI to refine my existing works, such as adjusting instrumentation, improving melodic lines, or polishing lyrics.

Interviewer: When using AI, do you tend to let it generate new content or help you optimize your existing ideas?

P05:I prefer using AI to optimize my existing ideas rather than asking it to generate an entire piece from scratch, because that would weaken my personal style.

Interviewer: Has AI helped you improve your learning efficiency or the quality of your work? In what way?

P05:Yes, it has greatly improved my work efficiency, especially during the revision stage. In the past, I might have needed several days to modify the instrumentation, but with AI assistance, I can now complete similar adjustments within a few hours.

Interviewer: How do you decide whether the information provided by AI is reliable or useful?

P05:I evaluate AI-generated content based on listening experience, musical theory, and my own professional judgment rather than accepting it blindly.

Interviewer: When using AI, do you feel that it stimulates your thinking or learning interest?

P05:Yes. I think AI can strongly stimulate my thinking during the creative process because it constantly provides unexpected suggestions or combinations of different styles.

Interviewer: How do you respond when AI provides answers that differ from your expectations?

P05:When the results do not match my expected direction, I do not immediately reject them. Instead, I analyze why AI created them in that way.

Interviewer: Do these unexpected results make you think more deeply about the problem?

P05:Yes. These unexpected results often inspire me to reconsider the relationship between melody and structure, and even reflect on my own creative habits.

Interviewer: Have you discovered new perspectives or connections as a result?

P05:Yes. Sometimes AI-generated music helps me notice rhythmic tension or balance issues in instrumentation that I had not paid attention to before, which leads to new creative ideas.

Interviewer: Do you feel particularly focused or immersed when using AI for learning?

P05:Yes. When composing with AI, I often enter a state of immersion. For example, when repeatedly adjusting AI generation parameters and listening to different versions, I sometimes lose track of time.

Interviewer: How does the learning experience with AI differ from traditional learning methods?

P05:Compared with traditional composition methods, AI’s immediate feedback makes it easier for me to maintain creative enthusiasm. It feels like I am “improvising” together with the system.

Interviewer: When AI’s response is unsatisfactory, do you give up or try to adjust it?

P05:I usually modify the input prompts or redefine the style labels rather than giving up directly.

Interviewer: Does this experience make your learning feel easier or more challenging?

P05:I think this interaction is both challenging and interesting. Although AI provides assistance, I still need to make the final decisions. This balance helps me stay focused.

Interviewer: After using AI, do you feel more confident in proposing creative ideas or solving problems?

P05:Yes. After using AI, I feel more confident trying new styles or techniques because AI can help me test whether my ideas are feasible.

Interviewer: Do you think AI has helped you improve your learning ability?

P05:Yes. It has improved my learning ability. For example, I can quickly understand the compositional characteristics of different musical styles.

Interviewer: Do you think AI makes you more willing to try new methods? Why?

P05:Yes. AI makes me more willing to try experimental music because even if an attempt does not work, I can quickly receive new inspiration and feedback.

Interviewer: Can you recall an experience with AI that made you feel, “I can actually do this”?

P05:Yes. Once, I wanted to imitate Debussy’s Impressionist style. AI helped me generate several harmonic progression samples, and I used them as a basis to rewrite and create a new piece. At that moment, I truly felt, “I can actually do this.”

Interviewer: When using AI, do you consider it a learning partner or collaborator? Why or why not?

P05:Yes. I see AI as a creative partner. It helps expand my thinking boundaries, while I am responsible for judging artistic value.

Interviewer: Through interacting with AI, have you rethought your learning methods or ideas?

P05:Yes. Through interacting with AI, I have reconsidered the meaning of “inspiration” in composition. Creativity is not only a sudden moment of inspiration; it can also be guided and generated through a process.

Interviewer: Do you modify or refine your ideas with AI’s help?

P05:Yes. I often adjust melodies, rhythms, or structural designs based on AI feedback, creating a cycle of continuous optimization and co-creation.

Interviewer: Do you reflect on AI’s limitations and your own role in the learning process?

P05:Yes. I also reflect on AI’s limitations. For example, its emotional expression is still not deep enough, which reminds me that human intuition and feelings remain irreplaceable parts of music.

Interviewer: Do you think using AI makes your learning outcomes more creative or of higher quality?

P05:Yes. I believe AI has improved the creativity and overall quality of my works, especially in terms of style integration and structural innovation.

Interviewer: Could you give an example of how AI has helped you improve your results?

P05:Once, I used AI to assist in creating a piece that combined electronic music and orchestral elements. The rhythmic layers suggested by AI greatly inspired me, and the final work won first prize in an academic competition.

Interviewer: Without AI, do you think you could have achieved similar learning outcomes?

P05:Yes, I could still complete my works without AI, but the process would not be as efficient, and it would be more difficult to break through the limitations of my existing style.

Interviewer: Do you think AI’s greatest contribution lies in “inspiring thinking” or “completing tasks”?

P05:I think AI’s greatest contribution is “inspiring thinking.” It is like a creative mirror that allows me to see possibilities I had not considered before, rather than simply helping me complete tasks.

**Participant: P06**

Interviewer: When using AI to complete learning tasks, do you try different approaches or new methods?

P06: Yes, I do. I often try different ways of asking questions or presenting input. For example, when writing a literature review, I ask AI to first generate perspectives from different schools of thought, and then guide it to compare or integrate these views. This process encourages me to think about problems more actively.

Interviewer: Do you use AI to explore knowledge or viewpoints that you were previously unfamiliar with?

P06: Yes, I often do. Especially in emerging topics in educational psychology, AI can quickly introduce me to research directions that I have not explored before, such as “generative AI-driven learning motivation models,” which helps me move beyond my original research framework.

Interviewer: Could you give an example of how AI has helped you “open up your thinking”?

P06:Once, I asked AI to analyze the relationship between “inquiry-based learning” and “reflective practice.” It explained the connection from a systems theory perspective, which inspired me to introduce a systems thinking perspective into my doctoral dissertation.

Interviewer: How is this experience of “exploration” different from your previous ways of learning?

P06:Previously, I relied more on literature searches and guidance from supervisors. AI allows me to stimulate thinking from multiple perspectives within a shorter time, making learning more interactive and providing immediate feedback.

Interviewer: Do you use AI to improve or refine your existing learning outcomes?

P06:Yes. I often use AI to polish paper abstracts or reorganize logical structures, which helps improve the clarity of my academic expression.

Interviewer: When using AI, do you tend to let it generate new content or help you optimize your existing ideas?

P06:I prefer to let it generate new content first and then combine it with my own ideas for further improvement. AI works like a brainstorming tool, but I am still responsible for integrating and selecting the useful parts.

Interviewer: Has AI helped you improve your learning efficiency or the quality of your work? In what way?

P06:Yes. For example, when writing academic papers, AI can help me quickly summarize literature and generate draft abstracts, allowing me to focus more on analysis and argument development.

Interviewer: How do you decide whether the information provided by AI is reliable or useful?

P06:I usually check the cited sources or compare the information with academic databases such as Web of Science. I see AI-generated information as a reference point rather than a final conclusion.

Interviewer: When using AI, do you feel that it stimulates your thinking or learning interest?

P06:Yes, very clearly. AI’s immediate feedback helps me maintain focus, especially when dealing with complex concepts. Its explanations often deepen my understanding.

Interviewer: How do you respond when AI provides answers that differ from your expectations?

P06:I do not reject them immediately. Instead, I try to analyze why AI responded in that way. In many cases, these “deviations” actually lead to new ways of thinking.

Interviewer: Do these unexpected results make you think more deeply about the problem?

P06:Yes. Especially when comparing educational theories, AI’s inconsistent responses sometimes encourage me to redefine the boundaries of the research problem.

Interviewer: Have you discovered new perspectives or connections as a result?

P06:Yes. For example, I found that the relationship between “generative AI” and “constructivist learning” was stronger than I originally thought, which changed my theoretical model.

Interviewer: Do you feel particularly focused or immersed when using AI for learning?

P06:Yes. Especially during multiple rounds of discussion with AI, I can enter a state of “research co-creation” flow.

Interviewer: How does the learning experience with AI differ from traditional learning methods?

P06:Traditional learning is more linear, while AI-based learning is dynamic and interactive, allowing me to continuously adjust my direction.

Interviewer: When AI’s response is unsatisfactory, do you give up or try to adjust it?

P06:I make adjustments. AI’s performance depends on the quality of my input, and this process of “dialogue and refinement” is also part of learning.

Interviewer: Does this experience make your learning feel easier or more challenging?

P06:It is more challenging because AI encourages me to constantly reflect on whether my input is accurate and precise. At the same time, it also contributes to my growth.

Interviewer: After using AI, do you feel more confident in proposing creative ideas or solving problems?

P06:Yes. AI helps me recognize gaps in my knowledge, and it also makes me believe that “by adjusting my thinking, I can develop innovative research.”

Interviewer: Do you think AI has helped you improve your learning ability?

P06:Yes, significantly. Especially in concept construction and interdisciplinary integration, AI provides efficient support.

Interviewer: Do you think AI makes you more willing to try new methods? Why?

P06:Yes, because the cost of failure becomes lower. AI provides immediate feedback, which gives me more confidence to experiment.

Interviewer: Can you recall an experience with AI that made you feel, “I can actually do this”?

P06:Yes. Once, I used AI to help me construct a preliminary model for my doctoral dissertation. The ideas it generated made me realize that I was also capable of conducting systematic theoretical integration.

Interviewer: When using AI, do you consider it a learning partner or collaborator? Why or why not?

P06:I think AI is more like an “assistant-type collaborator.” It cannot replace me, but it can help me advance my thinking.

Interviewer: Through interacting with AI, have you rethought your learning methods or ideas?

P06:Yes. I now pay more attention to the quality of questions and logical thinking rather than simply memorizing knowledge.

Interviewer: Do you modify or refine your ideas with AI’s help?

P06:Yes, especially when adjusting research frameworks or refining research questions.

Interviewer: Do you reflect on AI’s limitations and your own role in the learning process?

P06:Yes, I often do. AI is good at integrating information, but it lacks academic judgment. The final decisions still need to be made by humans.

Interviewer: Do you think using AI makes your learning outcomes more creative or of higher quality?

P06:Yes. AI allows me to connect different theoretical frameworks more quickly, which helps me generate new research propositions.

Interviewer: Could you give an example of how AI has helped you improve your results?

P06:When designing a questionnaire, I used AI to optimize the wording of the items, which helped improve the reliability and cultural adaptability of the scale.

Interviewer: Without AI, do you think you could have achieved similar learning outcomes?

P06:I could probably achieve them, but it would take much longer. AI lowers the barriers to exploration, but creativity still depends on individual thinking.

Interviewer: Do you think AI’s greatest contribution lies in “inspiring thinking” or “completing tasks”?

P06:I think it contributes more to inspiring thinking. It is like a mirror that helps me see new possibilities and identify gaps in my knowledge.

**Participant: P07**

Interviewer: When using AI to complete learning tasks, do you try different approaches or new methods?

P07: Yes. I like adjusting the way I ask questions and observing the different responses generated by AI. For example, I may use different tones, keywords, or disciplinary perspectives when asking questions. Sometimes, simply changing one word can help me discover another aspect of the problem.

Interviewer: Do you use AI to explore knowledge or viewpoints that you were previously unfamiliar with?

P07: Yes, I often do. My major is educational philosophy, but AI often recommends interdisciplinary literature, such as studies from cognitive science or sociology, which expands my theoretical perspective.

Interviewer: Could you give an example of how AI has helped you “open up your thinking”?

P07:When preparing a paper, I asked AI to explain the relationship between “educational equity” and “technology ethics.” It analyzed the issue from the perspective of “algorithmic bias,” which inspired me to transform an originally abstract ethical discussion into a more concrete issue related to technological practice.

Interviewer: How is this experience of “exploration” different from your previous ways of learning?

P07:Previously, learning was relatively linear and relied mainly on literature and suggestions from supervisors. AI allows me to ask questions and receive feedback immediately. This interactive exploration makes me more active and creative in learning.

Interviewer: Do you use AI to improve or refine your existing learning outcomes?

P07:Yes. I often use AI to polish paper abstracts, adjust paragraph logic, or help me find more precise academic expressions.

Interviewer: When using AI, do you tend to let it generate new content or help you optimize your existing ideas?

P07:I prefer combining both approaches. I first use AI to generate initial ideas and then ask it to analyze whether the structure is reasonable, so that I can further improve my research design.

Interviewer: Has AI helped you improve your learning efficiency or the quality of your work? In what way?

P07:Yes, the improvement is significant. AI can quickly organize complex information, allowing me to spend more time on deeper thinking rather than mechanical information searching.

Interviewer: How do you decide whether the information provided by AI is reliable or useful?

P07:I usually check whether the sources cited by AI actually exist and verify them through databases such as CNKI or Google Scholar. I maintain a critical attitude toward any conclusions generated by AI.

Interviewer: When using AI, do you feel that it stimulates your thinking or learning interest?

P07:Yes. AI can respond to my questions quickly, and this immediate feedback keeps my curiosity alive and encourages me to continue exploring complex issues.

Interviewer: How do you respond when AI provides answers that differ from your expectations?

P07:I do not reject them immediately. Instead, I think about what these differences reveal. In many cases, AI’s “mistakes” actually expose the blind spots in my own thinking.

Interviewer: Do these unexpected results make you think more deeply about the problem?

P07:Yes. AI’s different perspectives help me analyze issues from multiple angles, and sometimes they encourage me to look for original literature to verify the logic behind them.

Interviewer: Have you discovered new perspectives or connections as a result?

P07:Yes. For example, I discovered that “educational equity” could be studied together with “generative AI ethics.” This interdisciplinary connection helped me redefine the direction of my paper.

Interviewer: Do you feel particularly focused or immersed when using AI for learning?

P07:Yes. Especially when discussing theoretical frameworks or research models with AI, I can enter a highly focused state of “academic flow.”

Interviewer: How does the learning experience with AI differ from traditional learning methods?

P07:Traditional learning usually progresses at a slower pace, while AI provides high-density information exchange. It feels like an immediate brainstorming process that encourages me to continuously refine my thinking.

Interviewer: When AI’s response is unsatisfactory, do you give up or try to adjust it?

P07:I make adjustments. Usually, I reflect on whether my question was not clear enough and then reconstruct my input. This process itself is also a form of learning.

Interviewer: Does this experience make your learning feel easier or more challenging?

P07:It is more challenging. AI provides feedback very quickly, and I need to integrate and select information within a short period of time. This kind of “cognitive stretching” helps me grow quickly.

Interviewer: After using AI, do you feel more confident in proposing creative ideas or solving problems?

P07:Yes. AI provides a visual thinking space, making it easier for me to develop innovative ideas and giving me more confidence to try.

Interviewer: Do you think AI has helped you improve your learning ability?

P07:Yes. Especially in logical expression and academic writing, I have gradually improved the organization of my academic thinking through repeated practice with AI.

Interviewer: Do you think AI makes you more willing to try new methods? Why?

P07:Yes. AI’s interactivity reduces the risk of trying new methods. I can test whether an idea is feasible at any time without being afraid of making mistakes.

Interviewer: Can you recall an experience with AI that made you feel, “I can actually do this”?

P07:Yes. Once, I used AI to help me construct the theoretical model of my paper. It extracted core variables from different studies, which made me realize that I could also systematically integrate theories.

Interviewer: When using AI, do you consider it a learning partner or collaborator? Why or why not?

P07:I consider it a “collaborator.” It does not replace my thinking, but it can engage in continuous intellectual conversations with me.

Interviewer: Through interacting with AI, have you rethought your learning methods or ideas?

P07:It made me realize that high-quality learning is not only about obtaining answers, but also about learning how to ask better questions.

Interviewer: Do you modify or refine your ideas with AI’s help?

P07:Yes. Especially in research hypotheses and methodology sections, AI can help me express my ideas more precisely.

Interviewer: Do you reflect on AI’s limitations and your own role in the learning process?

P07:Yes. I believe AI’s knowledge boundaries remind me that genuine understanding comes from human thinking and reflection, rather than technological computation.

Interviewer: Do you think using AI makes your learning outcomes more creative or of higher quality?

P07:Yes. AI accelerates my thinking process and helps me construct new ideas in a more systematic way.

Interviewer: Could you give an example of how AI has helped you improve your results?

P07:AI once helped me compare different scholars’ definitions of the “humanistic nature of educational technology.” This allowed me to propose a new conceptual framework in my paper.

Interviewer: Without AI, do you think you could have achieved similar learning outcomes?

P07:Perhaps I could, but it would take more time. AI allows me to complete the process of knowledge accumulation and reflection more quickly.

Interviewer: Do you think AI’s greatest contribution lies in “inspiring thinking” or “completing tasks”?

P07:Definitely “inspiring thinking.” It does not help me simply “finish tasks”; instead, it allows me to see “what else I can think about.”

**Participant: P08**

Interviewer: When using AI to complete learning tasks, do you try different approaches or new methods?

P08: Yes. My research involves experimental data analysis and model simulation, and AI allows me to try multiple analytical approaches. For example, I ask AI to help me compare different statistical methods and determine which one is more suitable for my data structure.

Interviewer: Do you use AI to explore knowledge or viewpoints that you were previously unfamiliar with?

P08: Yes, frequently. AI helps me quickly understand interdisciplinary knowledge. For example, when I study material properties, it recommends ideas related to physical chemistry or computational modeling, which helps me expand my research scope.

Interviewer: Could you give an example of how AI has helped you “open up your thinking”?

P08:When writing the introduction of a paper, I asked AI to summarize research trends in the application of a certain technology across different countries. It not only provided new perspectives but also made me realize that the research could be approached from the perspective of “sustainable development,” which was a new direction for me.

Interviewer: How is this experience of “exploration” different from your previous ways of learning?

P08:Previously, I relied on literature searches, but the efficiency was low. AI can provide ideas immediately and help me form hypotheses. This instant feedback makes research more creative and interactive.

Interviewer: Do you use AI to improve or refine your existing learning outcomes?

P08:Yes. I often ask AI to check whether the logic of my data analysis is rigorous or to optimize the structure of experimental reports, making them more consistent with academic standards.

Interviewer: When using AI, do you tend to let it generate new content or help you optimize your existing ideas?

P08:I prefer optimizing existing outcomes. AI is good at helping me clarify my thoughts or express complex results in a clearer way.

Interviewer: Has AI helped you improve your learning efficiency or the quality of your work? In what way?

P08:The improvement is very obvious. Especially in literature organization, result summarization, and English writing, AI saves me a great deal of time and improves the quality of my outcomes.

Interviewer: How do you decide whether the information provided by AI is reliable or useful?

P08:I compare AI’s answers with information from academic databases such as Web of Science and Scopus. At the same time, I check logical consistency and data sources to avoid fabricated citations.

Interviewer: When using AI, do you feel that it stimulates your thinking or learning interest?

P08:Yes. AI’s responses often bring new perspectives, such as suggesting variables that I have not considered before. This helps me maintain active thinking and enthusiasm for learning.

Interviewer: How do you respond when AI provides answers that differ from your expectations?

P08:I analyze the reasons. Sometimes, although AI’s answer is “wrong,” it encourages me to reflect on whether there are biases in my experimental assumptions.

Interviewer: Do these unexpected results make you think more deeply about the problem?

P08:Yes. They encourage me to examine problems from different perspectives and sometimes even guide me to design new experiments for verification.

Interviewer: Have you discovered new perspectives or connections as a result?

P08:Yes. For example, through AI, I discovered that ideas for optimizing the structure of certain materials could draw on principles of biological bionics. This helped me combine natural science with engineering.

Interviewer: Do you feel particularly focused or immersed when using AI for learning?

P08:Yes. When I continuously discuss experimental plans or paper structures with AI, I often enter a highly focused state.

Interviewer: How does the learning experience with AI differ from traditional learning methods?

P08:Traditional methods are relatively slow and fragmented, while AI’s immediate feedback allows me to continuously reason and think. It feels more like communicating with a “virtual research assistant.”

Interviewer: When AI’s response is unsatisfactory, do you give up or try to adjust it?

P08:I make adjustments. I change the way I provide input, such as adding more specific background information or constraints. Usually, this helps me obtain more accurate results.

Interviewer: Does this experience make your learning feel easier or more challenging?

P08:Both. While AI improves efficiency, it also requires me to make more active judgments and filter information, which actually increases my cognitive load.

Interviewer: After using AI, do you feel more confident in proposing creative ideas or solving problems?

P08:Yes. AI helps me quickly verify the rationality of hypotheses, giving me more confidence when proposing new ideas.

Interviewer: Do you think AI has helped you improve your learning ability?

P08:The improvement is significant. I have learned to express problems in a more systematic way and better integrate complex information.

Interviewer: Do you think AI makes you more willing to try new methods? Why?

P08:Yes. AI reduces the cost of experimentation. For example, I can quickly test different model parameters, and this immediate feedback encourages me to continue experimenting.

Interviewer: Can you recall an experience with AI that made you feel, “I can actually do this”?

P08:Yes. Once, AI helped me build a prediction model for experimental results. After verification, I found that its accuracy was very high, which made me realize that I was fully capable of integrating AI into scientific research.

Interviewer: When using AI, do you consider it a learning partner or collaborator? Why or why not?

P08:Yes. AI is like a “tireless research assistant” that I can communicate with at any time. It helps me explore and evaluate different approaches.

Interviewer: Through interacting with AI, have you rethought your learning methods or ideas?

P08:Yes. Previously, I pursued a single correct answer, but now I focus more on process-oriented thinking and multi-path verification.

Interviewer: Do you modify or refine your ideas with AI’s help?

P08:Yes. Especially when writing papers, AI helps me clarify the logic of my arguments and make my research more rigorous.

Interviewer: Do you reflect on AI’s limitations and your own role in the learning process?

P08:Yes. I clearly understand that AI is an auxiliary tool rather than the subject of thinking. My role is to judge, integrate, and innovate, rather than passively accept its outputs.

Interviewer: Do you think using AI makes your learning outcomes more creative or of higher quality?

P08:Yes. AI helps me integrate different theories into experiments, improving the originality of my research.

Interviewer: Could you give an example of how AI has helped you improve your results?

P08:AI once helped me reorganize the logical structure of my paper, making my research ideas more coherent. As a result, reviewers considered the paper to have a “clear structure and solid arguments.”

Interviewer: Without AI, do you think you could have achieved similar learning outcomes?

P08:I might eventually achieve the same outcomes, but it would be slower and less efficient. AI accelerates the process of knowledge processing.

Interviewer: Do you think AI’s greatest contribution lies in “inspiring thinking” or “completing tasks”?

P08:It contributes more to “inspiring thinking.” It does not replace researchers; instead, it expands my thinking boundaries.
