# Supplementary material for: How Generative Artificial Intelligence (GAI) reshapes students’ creativity in higher education: A chain path modeling Study from “Exploration–Exploitation” to “Co-Creation–Reflection”
Source: PLoS One. 2026 Sep 24;21(9):e0358186. doi: 10.1371/journal.pone.0358186 (PMC13606950; doi:10.1371/journal.pone.0358186)
Supplement: Supplementary file 2 [file pone.0358186.s003.docx]

**Appendix A**

Measurement Items and Sources

| Model Building | code | Questionnaire Questions | Literature Sources |
| --- | --- | --- | --- |
| Basic Information | Q1 | Gender:☐ Male ☐ Female ☐ Other | |
|  | Q2 | Identity:□ Undergraduate student □ Master's student □ Doctoral student | |
|  | Q3 | Duration of using generative AI:□ Less than 3 months □ 3–6 months □ 6–12 months □ 1–2 years □ More than 2 years | |
|  | Q4 | Commonly used GAI tools (select all that apply):□ ChatGPT □ Wenxin Yiyan (Ernie Bot) □ Deepseek □ Doubao □ Other: ______ | |
| Exploration | Q5 | I actively try to use GAI to explore new ideas and creativity. | March，1991 |
|  | Q6 | During the learning process, I use GAI tools to pose novel questions. |  |
|  | Q7 | I often explore different functions of GAI tools to discover new uses. |  |
|  | Q8 | When using GAI, I tend to try unprecedented methods to solve problems. |  |
|  | Q9 | I am willing to invest time to discover new areas of knowledge within GAI tools. |  |
| Exploitation | Q10 | I tend to use GAI to complete learning tasks I am already familiar with. | March，1991 |
|  | Q11 | I use GAI tools to improve task efficiency rather than to seek new ideas. |  |
|  | Q12 | I rely on GAI technologies to enhance existing solutions. |  |
|  | Q13 | I apply GAI to refine methods that have worked for me in the past. |  |
|  | Q14 | I mostly reuse familiar GAI functions to complete tasks. |  |
| Co-Creation | Q15 | I am able to co-create creative content in collaboration with GAI. | Hwang & Lee, 2025；McGuire et al., 2024 |
|  | Q16 | I perceive GAI as a collaborative partner in my creative process, rather than just a tool. |  |
|  | Q17 | I believe that co-creating with GAI inspires me to generate new and valuable ideas. |  |
|  | Q18 | In creative tasks, my contributions and GAI’s input complement each other. |  |
|  | Q19 | GAI’s involvement helps me better solve complex creative problems. |  |
| Reflection | Q20 | I reflect on the learning process assisted by GAI. | Phan, 2009 |
|  | Q21 | I analyze how GAI influences my thinking during collaboration. |  |
|  | Q22 | After using GAI, I summarize the reasons behind successes and failures. |  |
|  | Q23 | I evaluate whether the ideas generated with GAI have practical value. |  |
|  | Q24 | I consider how GAI changes my learning strategies. |  |
| Perceived Cognitive Stimulation | Q25 | Using GAI prompts me to think more deeply. | Sun & Rueda, 2012 |
|  | Q26 | GAI's responses encourage me to understand problems from different perspectives. |  |
|  | Q27 | GAI helps me form new cognitive connections in the learning process. |  |
|  | Q28 | GAI encourages me to engage in critical thinking. |  |
|  | Q29 | Interacting with GAI promotes my re-conceptualization of problems. |  |
| Flow Experience | Q30 | When using GAI, I often enter a state of "flow" or deep immersion. | Csikszentmihalyi & Csikzentmihaly, 1990 |
|  | Q31 | I feel highly focused when interacting with GAI. |  |
|  | Q32 | I often lose track of time while using GAI. |  |
|  | Q33 | Collaborating with GAI puts me in a state of full concentration. |  |
|  | Q34 | Using GAI allows me to become fully engaged in the learning task. |  |
| Self-efficacy in Creativity | Q35 | I believe I can use generative AI (GAI) to develop creative solutions. | Tierney & Farmer, 2002;Beghetto, 2006 |
|  | Q36 | Even when tasks are challenging, I can come up with novel ideas with the help of GAI. |  |
|  | Q37 | I am able to flexibly use the knowledge provided by GAI to generate new ideas. |  |
|  | Q38 | I have the ability to produce unique creative outcomes using GAI. |  |
|  | Q39 | I am confident that I can perform well in creative tasks assisted by GAI. |  |
| Student Creativity | Q40 | I can propose distinctive learning ideas or solutions with the assistance of GAI. | Zhou & George, 2001;Kaufman, 2016 |
|  | Q41 | My GAI-assisted work often demonstrates unique forms of expression. |  |
|  | Q42 | I am able to integrate knowledge from different domains provided by GAI into new ideas. |  |
|  | Q43 | I often try to complete assignments using novel approaches suggested by GAI. |  |
|  | Q44 | I believe that the works produced with GAI-assisted learning are creative. |  |

**Appendix B**

Interview Items and Sources

| Model Building | code | Questionnaire Questions | Purpose | Literature Sources |
| --- | --- | --- | --- | --- |
| Basic Information | Q1 | Gender:☐ Male ☐ Female ☐ Other | | |
|  | Q2 | Identity:□ Undergraduate student □ Master's student □ Doctoral student | | |
|  | Q3 | Duration of using generative AI:□ Less than 3 months □ 3–6 months □ 6–12 months □ 1–2 years □ More than 2 years | | |
|  | Q4 | Commonly used GAI tools (select all that apply):□ ChatGPT □ Wenxin Yiyan (Ernie Bot) □ Deepseek □ Doubao □ Other: ______ | | |
| Exploration | Q5 | When using AI to complete learning tasks, do you try different approaches or new methods? | To explore whether students use AI to discover new ideas, methods, or perspectives. | March，1991 |
|  | Q6 | Do you use AI to explore knowledge or viewpoints that you were previously unfamiliar with? |  |  |
|  | Q7 | Could you give an example of how AI has helped you “open up your thinking”? |  |  |
|  | Q8 | How is this experience of “exploration” different from your previous ways of learning? |  |  |
| Exploitation | Q9 | Do you use AI to improve or refine your existing learning outcomes? | To understand how students apply AI to refine or enhance their existing knowledge and skills. |  |
|  | Q10 | When using AI, do you tend to let it generate new content or help you optimize your existing ideas? |  |  |
|  | Q11 | Has AI helped you improve your learning efficiency or the quality of your work? In what way? |  |  |
|  | Q12 | How do you decide whether the information provided by AI is reliable or useful? |  |  |
| Perceived Cognitive Stimulation | Q13 | When using AI, do you feel that it stimulates your thinking or learning interest? | To investigate whether AI stimulates students’ learning interest or cognitive engagement. | Sun & Rueda, 2012 |
|  | Q14 | How do you respond when AI provides answers that differ from your expectations? |  |  |
|  | Q15 | Do these unexpected results make you think more deeply about the problem? |  |  |
|  | Q16 | Have you discovered new perspectives or connections as a result? |  |  |
| Flow Experience | Q17 | Do you feel particularly focused or immersed when using AI for learning? | To understand the level of engagement and “flow” students experience during AI-assisted learning. | Csikszentmihalyi & Csikzentmihaly, 1990 |
|  | Q18 | How does the learning experience with AI differ from traditional learning methods? |  |  |
|  | Q19 | When AI’s response is unsatisfactory, do you give up or try to adjust it? |  |  |
|  | Q20 | Does this experience make your learning feel easier or more challenging? |  |  |
| Self-Efficacy in Creativity | Q21 | After using AI, do you feel more confident in proposing creative ideas or solving problems? | To examine whether AI enhances students’ confidence in their creativity or problem-solving abilities. | Tierney & Farmer, 2002;Beghetto, 2006 |
|  | Q22 | Do you think AI has helped you improve your learning ability? |  |  |
|  | Q23 | Do you think AI makes you more willing to try new methods? Why? |  |  |
|  | Q24 | Can you recall an experience with AI that made you feel, “I can actually do this”? |  |  |
| Co-creation & Reflection | Q25 | When using AI, do you consider it a learning partner or collaborator? Why or why not? | To understand how students collaborate with AI and reflect on their learning processes. | Hwang & Lee, 2025；McGuire et al., 2024；Phan, 2009 |
|  | Q26 | Through interacting with AI, have you rethought your learning methods or ideas? |  |  |
|  | Q27 | Do you modify or refine your ideas with AI’s help? |  |  |
|  | Q28 | Do you reflect on AI’s limitations and your own role in the learning process? |  |  |
| Student Creativity | Q29 | Do you think using AI makes your learning outcomes more creative or of higher quality? | To explore the actual impact of AI on the innovativeness and quality of students’ learning outcomes. | Zhou & George, 2001;Kaufman, 2016 |
|  | Q30 | Could you give an example of how AI has helped you improve your results? |  |  |
|  | Q31 | Without AI, do you think you could have achieved similar learning outcomes? |  |  |
|  | Q32 | Do you think AI’s greatest contribution lies in “inspiring thinking” or “completing tasks”? |  |  |
